# Genetic dissection of sorghum grain quality traits using diverse and segregating populations

**DOI:** 10.1007/s00122-016-2844-6

**Published:** 2016-12-27

**Authors:** Richard E. Boyles, Brian K. Pfeiffer, Elizabeth A. Cooper, Bradley L. Rauh, Kelsey J. Zielinski, Matthew T. Myers, Zachary Brenton, William L. Rooney, Stephen Kresovich

**Affiliations:** 1 Department of Genetics and Biochemistry, Clemson University, Clemson, SC 29634 USA; 2 Advanced Plant Technology Program, Clemson University, Clemson, SC 29634 USA; 3 Department of Soil and Crop Sciences, Texas A&M University, 2474 TAMU, College Station, TX 77843 USA; 4 Plants for Human Health Institute, North Carolina State University, Kannapolis, NC 28081 USA; 5 Institute of Translational Genomics, Clemson University, Clemson, SC 29634 USA

## Abstract

**Key message:**

**Coordinated association and linkage mapping identified 25 grain quality QTLs in multiple environments, and fine mapping of the** ***Wx*** **locus supports the use of high-density genetic markers in linkage mapping.**

**Abstract:**

There is a wide range of end-use products made from cereal grains, and these products often demand different grain characteristics. Fortunately, cereal crop species including sorghum [*Sorghum bicolor* (L.) Moench] contain high phenotypic variation for traits influencing grain quality. Identifying genetic variants underlying this phenotypic variation allows plant breeders to develop genotypes with grain attributes optimized for their intended usage. Multiple sorghum mapping populations were rigorously phenotyped across two environments (SC Coastal Plain and Central TX) in 2 years for five major grain quality traits: amylose, starch, crude protein, crude fat, and gross energy. Coordinated association and linkage mapping revealed several robust QTLs that make prime targets to improve grain quality for food, feed, and fuel products. Although the amylose QTL interval spanned many megabases, the marker with greatest significance was located just 12 kb from *waxy* (*Wx*), the primary gene regulating amylose production in cereal grains. This suggests higher resolution mapping in recombinant inbred line (RIL) populations can be obtained when genotyped at a high marker density. The major QTL for crude fat content, identified in both a RIL population and grain sorghum diversity panel, encompassed the *DGAT1* locus, a critical gene involved in maize lipid biosynthesis. Another QTL on chromosome 1 was consistently mapped in both RIL populations for multiple grain quality traits including starch, crude protein, and gross energy. Collectively, these genetic regions offer excellent opportunities to manipulate grain composition and set up future studies for gene validation.

**Electronic supplementary material:**

The online version of this article (doi:10.1007/s00122-016-2844-6) contains supplementary material, which is available to authorized users.

## Introduction

Sorghum [*Sorghum bicolor* (L.) Moench] is a subsistence crop across the semi-arid tropics in sub-Saharan Africa and Southern Asia, where the grain is a dietary staple for an estimated half billion people (National Research Council 1996). Furthermore, grain sorghum is widely produced on marginal lands in Australia and the United States, where it is primarily grown for animal feed and ethanol conversion (Wang et al. 2008; Mace and Jordan 2011). Grain sorghum, like other cereal crops, can also be utilized to create additional end-use products. Sorghum grain has been used to make baking flours, pop sorghum, alcoholic beverages, pet foods, and packaging materials (Fang and Hanna 2000; Udachan et al. 2012; Zhu 2014). These various products can require different grain characteristics and thus can alter crop ideotype. Identifying genes influencing sorghum grain composition would help manipulate grain texture and quality to accommodate existing end-use markets and promote new product development (Bean et al. 2016). In addition, understanding the chemical and genetic components underlying the gross energy content of sorghum would enable breeders to increase the overall feed efficiency when the grain is grown for livestock feed through selective breeding and trait introgression.

The traditional selection schema implemented by plant breeders throughout history has justifiably focused primarily on yield and stress resistance (Morris and Sands 2006). Priority must first be given to developing hybrids and cultivars capable of producing for the farmer. However, a global malnutrition crisis has shifted emphasis toward improving the grain quality of staple cereal crops and ensuring food security (Rosegrant and Cline 2003), and significant investments have been made to address these concerns (Varmus et al. 2003). Additionally, the livestock industry is continuously searching for agricultural products that accelerate growth and enhance the nutritional quality of their animals (Cowieson 2005; Kriegshauser et al. 2006; Smith et al. 2015). Animals consume approximately one-third of worldwide grain production (Pimentel et al. 1997), substantiating the need for improved grain products for this end-use. Preferred grain composition varies depending on end-use, and unlocking the network of genes regulating grain quality traits will help facilitate the manipulation of macronutrient content and digestibility for plant breeders. The first critical component is identifying genes or gene regions useful for sorghum biofortification that do not hinder its agronomic yield or productivity. Results in wheat found that improvement of certain grain quality parameters do not lead to a decrease in grain yield (Anderson et al. 1997). Additionally, Jampala et al. (2012) evaluated sorghum recombinant inbred lines (RILs) segregating for multiple grain quality traits and found several high quality grain lines to be among the top yielding.

Cereal crops, including sorghum, produce grains rich in carbohydrates, primarily starch. Cereals also store considerable concentrations of protein and fat in the caryopsis to support embryogenesis (Hubbard et al. 1950), which can be energy-rich and provide essential nutrients needed for adequate growth and development of both humans and animals. However, digestibility of these macronutrients, particularly starch and protein, can vary widely among different grain sorghums (Rooney and Pflugfelder 1986; Axtell et al. 1981; Sang et al. 2008). Therefore, gross energy, the amount of heat generated during combustion, was evaluated in this study as an estimate of sorghum digestibility because a strong positive correlation between gross energy and digestible energy was previously identified in cereals (Bhatty and Wu 1974). Additionally, specific sorghum genotypes contain high levels of polyphenols that have antioxidant properties and other potential health benefits (Rhodes et al. 2014). Sorghum was shown to have extensive variation for several grain quality traits including the three primary macronutrients (starch, protein, and fat) across diverse germplasm (Shewayrga et al. 2012; Sukumaran et al. 2012; Rhodes et al. 2016), which gives promise to advance sorghum biofortification and breeding for specific end-use products.

There have been numerous genetic studies on grain quality traits across the major cereal crops including maize (Séne et al. 2000; Wilson et al. 2004; Cook et al. 2012), rice (He et al. 1999; Aluko et al. 2004; Li et al. 2004), sorghum (Ibrahim et al. 1985; Rami et al. 1998; Sukumaran et al. 2012; Rhodes et al. 2016), and wheat (Huang et al. 2006; McCartney et al. 2006). The starch biosynthesis pathway in maize has been well described (Séne et al. 2000; Whitt et al. 2002), and the major genes involved in maize starch biosynthesis are highly conserved in sorghum for valuable comparative analyses (Table S1). It is in the final steps of this pathway where starch synthases and starch branching and debranching enzymes work collectively to determine the ratio of amylose to amylopectin, the two major components of starch. Low amylose sorghums, better known as waxy sorghums, have been shown to have increased feed and ethanol conversion efficiency compared to normal, non-waxy genotypes (Sherrod et al. 1969; Yan et al. 2011). Recent analysis of whole genome resequencing data across sorghum accessions indicated many of the starch biosynthesis genes are under selection in historic cultivars (Campbell et al. 2016).

Grain storage protein and fat biosynthesis pathways are less characterized in cereals, but genome-wide association studies (GWAS) in maize have revealed multiple candidate genes for each macronutrient (Cook et al. 2012; Li et al. 2013). Generally, the majority of amino acids within sorghum grains are stored in protein bodies, called kafirins, which are similar to zeins in maize (Saito et al. 2012). Kafirins are located in the endosperm and can form a tight matrix with starch granules to reduce both protein and starch digestibility, which lowers feed efficiency (Duodu et al. 2003). Loss-of-function mutants of *floury*-*2* and *opaque*-*2*, major genes regulating kafirin levels and protein digestibility, were identified by Singh and Axtell (1973), and these mutants contain high lysine levels compared to normal genotypes. Although crude fat, or ether-extracted lipids, has the lowest concentration (2–4% dry matter) of macronutrients in sorghum grain, its high calorie density makes the trait a valid target to increase sorghum nutritional value for the animal feed industry (Kriegshauser et al. 2006). Of the identified major effect genes compiled by Mace and Jordan (2010), none were connected to fat biosynthesis. This reinforces that genetic dissection of this trait would be of value.

In an effort to identify rare and major effect loci through multiple mapping strategies, this research included a diverse association panel as well as two RIL populations. This multi-population approach was designed to combine the statistical resolution of a diversity panel with the statistical power (high allele frequency) of segregating RILs. Combining populations also enables detection of both additive and dominance effects. Parental lines used to develop the RILs had contrasting protein and starch digestibility among other grain quality traits (Miller et al. 1992; Weaver et al. 1998; Harris et al. 2007; Jampala et al. 2012). Transgressive segregation for additional traits such as starch and protein content resulted in large variation present within each population although parental lines contained similar trait values. In addition, the association panel contained greater variation than the biparental families for most quality traits, suggesting favorable alleles that have yet to be utilized for grain quality improvement exist in diverse germplasm. Loci containing these favorable alleles were mapped to different locations across the genome to understand the genetic basis of macronutrient content as well as the gross energy value generated from the ratio and interactions of these macronutrients. Identifying these grain quality loci could allow breeding efforts to develop elite grain sorghums that contain enhanced properties for food, feed, and biofuel end-uses without compromising productivity.

## Materials and methods

### Grain sorghum diversity panel

A total of 390 diverse grain accessions were planted in 2013 and 2014 in Florence, South Carolina. The majority (*n* = 332) of the 390 accessions within the grain sorghum diversity panel (GSDP) were in the original U.S. sorghum association panel (Casa et al. 2008), and the additional accessions were included because they have a historical relevance, diverse origin, or distinctive phenotype. Experimental field design parameters are fully described in Boyles et al. (2016). Briefly, the experiment was planted 15 May 2013 and 7 May 2014 in a 2× replicated randomized complete block design with plot dimensions of two rows, 6.1 m length, and a row spacing of 0.762 m. Plant density was approximately 130,000 plants ha^−1^ and plots were irrigated as needed to characterize sorghum grain quality traits in favorable environments. No fungicides were applied in the GSDP to control grain mold (*Fusarium* spp.) and anthracnose (*Colletotrichum sublineolum*) populations.

### Recombinant inbred line populations

Two biparental RIL populations segregating for grain quality traits were also studied to compare linkage mapping with association mapping in diverse sorghum germplasm. Both populations share a common parent, BTxARG-1, which has a white pericarp color, waxy endosperm (low amylose), and additional qualities that make it an attractive parental line for food grade hybrid development (Miller et al. 1992). The other parents were P850029, a highly digestible protein breeding line with high lysine content (Weaver et al. 1998; Jampala et al. 2012), and BTx642, a yellow pericarp sorghum with post-flowering drought resistance (Rosenow et al. 2002; Harris et al. 2007). Both populations were phenotyped in the F_4:5_ generation and DNA from tissue of F_5_ plants was genotyped. Populations, BTx642/BTxARG-1 and BTxARG-1/P850029, are hereafter referenced according to their unique parents, BTx642 and P850029. BTx642 contained 191 individuals and P850029 consisted of 279 lines with quality genotyping data.

Populations were planted with an Almaco cone planter in a 2× replicated randomized complete block design across two years (2014 and 2015) in Blackville, SC and College Station, TX. These experiments are hereafter referred to as SC14, SC15, TX14, and TX15. The SC experiments were planted between 4 and 15 May, depending on year and population, with replicates always planted on the same day. Individual plots in SC14 and SC15 consisted of 6.1 m single row plots with a row spacing of 0.965 m, with the exception of the BTx642 population in SC14, which contained plot lengths of 3.05 m as a result of seed limitations. Agronomic practices for SC14 and SC15 were similar to those of the GSDP except that only 60 units of lay-by N was applied prior to anthesis because a legume crop (SC14: peanut, SC15: soybean) was planted the prior year to provide an additional N source. In the SC14 experiment, the field was cultivated 44 days after planting (DAP) to reduce grass weed pressure. To control for headworms, 0.36 L ha^−1^ of Endigo ZC (pyrethroid) in SC14 and 1.2 L ha^−1^ of Prevathon (chlorantraniliprole) in SC15 were applied during the grain filling stage. In SC15, two applications of Transform WG (sulfoxaflor) at 0.1 L ha^−1^ were administered 3 weeks apart to reduce plant stress from sugarcane aphids (*Melanaphis saccari*). Additionally, a fungicide treatment (1 L ha^−1^ Quilt Xcel) was applied approximately 100 DAP in both SC14 and SC15 to reduce the confounding effect of biotic damage on grain quality.

The TX14 and TX15 experiments were planted on 21 April and 8 April, respectively. Plots were planted using John Deere Max-Emerge II units. The TX14 experiment was planted following a soybean crop in the previous growing season while the TX15 experiment followed cotton. The plots in the TX environment were 5.5 m long with row spacing of 0.762 m. In 2014, each plot consisted of one row; in 2015, the experiment was grown as two-row plots. Both TX environments had supplemental irrigation available. Due to above average summer rainfall, irrigation was not applied during the TX14 growing season. The TX15 experiment was flood irrigated once on 10 July. Both TX environments were grown with ridge till cultivation. Rolling cultivation was employed twice in the 2015 season to control heavy weed pressure. Plots in TX14 were fertilized with 15 units N and 52 units P prior to planting. Slightly higher pre-plant fertilizer rates of 22 units N and 66 units P were required in TX15. Approximately 80 and 60 units of lay-by N (UAN) were, respectively, administered in 2014 and 2015. Pre- and post-emergent herbicide applications in both years were similar to those of the SC experiments.

### Agronomic traits and grain processing

Number of days to anthesis was recorded for each plot when approximately 50% of plants reached mid-bloom. Plant height was measured from the ground to the apex of the main panicle from a representative plant in each plot. For the GSDP and RIL experiments in SC, three random panicles were harvested per plot at physiological maturity. The first and last plants in each row were not harvested to eliminate confounding results caused by border effect. For the RIL experiment in the TX location, ten representative panicles were harvested at random from each plot at post-physiological maturity (mid-August). Harvested panicles from both environments were dried to constant moisture and subsequently hand-threshed. Threshed grain in SC was run through an air aspirator (AT Ferrell Company, Inc.) and then through a wheat dehuller (Precision Machine Co., Inc.) to remove glumes and other plant debris. Grain from the TX location was cleaned using a Wintersteiger LD18 (Wintersteiger Ag). A 25 g homogenized subsample of grain was ground to 1-mm particle size with a CT 193 Cyclotec Sample Mill (FOSS North America) prior to compositional analysis. Generally, near-infrared spectroscopy (NIRS) performs better on ground versus whole grain samples in sorghum (de Alencar Figueirido et al. 2010) as pericarp thickness has been shown to affect whole grain NIRS predictability (Guindo et al. 2016).

### Grain quality phenotypes

Near-infrared spectroscopy was used to analyze grain composition traits simultaneously. Cereal grain quality traits including the five evaluated in this study have been previously measured with high predictability using NIRS (Kays and Barton 2002; de Alencar Figueirido et al. 2010). Ground grain was evenly placed in a 43 mL Teflon dish that gradually rotated during NIRS analysis to improve sampling accuracy. All traits were measured with a DA7250 NIR analyzer (Perten Instruments). For initial calibration of the Perten DA7250, wet chemistry was performed on a subset of 100 samples within the GSDP. The wet chemistry was performed at Dairyland Laboratories, Inc. (Arcadia, WI) and the Quality Assurance Laboratory at Murphy-Brown, LLC (Warsaw, NC). The Quality Assurance Laboratory also estimated gross energy using bomb calorimetry. Existing calibrations of amylose, crude fat, and starch content were improved with quantification of 35 (15 unique and ten blind duplicates) P850029 samples from SC15 performed at Dairyland Laboratories, Inc. The samples were chosen based on extreme values in SC15 that fell outside the existing calibration curve. Final calibration equations resulted in *r* ^*2*^ values of 0.691 for amylose (% starch), 0.893 for starch (% dry basis), 0.964 for crude protein (% dry basis), 0.554 for crude fat (% dry basis), and 0.883 for gross energy (kcal kg^−1^).

### Statistical analysis

Trait variability, correlations, and mapping were calculated using replicate means within each year and location. The cor() and cor.test() functions in R software (R Core Development Team 2015) were used to generate Pearson pairwise correlation coefficients and determine significance. Broad-sense heritability (*H* ^*2*^) estimates were calculated from variance components generated with the “lme4” R package as described in Boyles et al. (2016). All components were treated as random effects. For the RIL populations, replicates were nested in location-by-year interaction as shown below:

$${\text{GSDP}} .\quad Trait \sim G + Y + R \times Y + G \times R + G \times Y,$$

$${\text{RIL}} .\quad Trait \sim G + L + Y + R\% in\% L \times Y + G \times L + G \times Y,$$

where *G* is genotype, *L* is location, *R* is replication, and *Y* is year. Multiplication symbols indicate interactions between variables while nesting is denoted by “%*in*%”.

Heritability was calculated across populations using the following equations:

$${\text{GSDP}}.\quad H^{2} = \frac{{\sigma_{G}^{2} }}{{\left[ { \sigma_{G}^{2} + \left( {\frac{{\sigma_{GxR}^{2} }}{R}} \right) + \left( {\frac{{\sigma_{GxY}^{2} }}{Y}} \right) + \left( {\frac{{\sigma_{E}^{2} }}{RY}} \right)} \right]}},$$

$${\text{RILs}}.\quad H^{2} = \frac{{\sigma_{G}^{2} }}{{\left[ { \sigma_{G}^{2} + \left( {\frac{{\sigma_{GxL}^{2} }}{L}} \right) + \left( {\frac{{\sigma_{GxY}^{2} }}{Y}} \right) + \left( {\frac{{\sigma_{E}^{2} }}{LY}} \right)} \right]}},$$

where *G* is genotype, *L* is location, *R* is replication, *Y* is year, and *E* is error.

Pairwise linkage disequilibrium (LD) between SNPs was generated using the R package “genetics” (Warnes et al. 2012). Resulting LD values were grouped based on physical distance between SNP pairs into 100 bp windows, and the average LD for each window was plotted to determine the genome-wide pattern of LD decay for each population. Chromosome-wise recombination fractions for the two RIL populations were produced using the “qtl” (R/qtl) package in R (Broman et al. 2003).

Phenotypic variance explained (PVE) for each associated SNP from the grain quality GWAS was estimated with the Genome Association and Prediction Integrated Tool (GAPIT) package in R. In the RIL populations, genetic variance explained by individual quantitative trait loci (QTLs) was calculated based on the maximum LOD score within the quantitative trait locus (QTL) interval as follows: 1–10^−2 LOD/*n*^, where LOD is the LOD score produced from the R/qtl scanone() function and *n* is the number of RILs included in the QTL analysis (Broman et al. 2003). To determine PVE by each QTL, the genetic variance explained was multiplied by the overall broad-sense heritability (Broman et al. 2003): PVE = *H* ^*2*^ (1–10^−2 LOD/*n*^).

### Genotype-by-sequencing (GBS)

Genotyping for the GSDP has been thoroughly described in Morris et al. (2013) and Boyles et al. (2016). To allow for comparison to associations found in the RIL populations, the raw data reads of the GSDP were realigned to the new *Sorghum bicolor v3.1* reference genome (https://phytozome.jgi.doe.gov/), and SNPs were re-called using the previously described pipelines (Morris et al. 2013; Boyles et al. 2016). A total of 268,896 SNPs were used in GWAS that passed the minor allele frequency (MAF) cutoff of 0.05. Of the 390 accessions, a total of 368 and 378 contained quality genetic and phenotypic data for GWAS in 2013 and 2014, respectively.

For the RIL populations, single plant leaf tissue from each individual RIL and each parent (BTxARG-1, BTx642, and P850029) was harvested from 2-week-old seedlings. Plant tissue was lyophilized and sent to the Cornell University Genomic Diversity Facility for genotyping. Individual DNAs were extracted using the CTAB protocol (Mace et al. 2003) and digested with the restriction enzyme ApeKI. Digested DNA fragments of 96 individuals were ligated to a unique barcode adaptor and subsequently pooled for sequencing. Five 96-plex GBS libraries were single-end sequenced using an Illumina HiSeq 2500 to obtain 64-bp reads (excluding adaptor sequences). Reads were aligned, and SNPs were both called and imputed with the TASSEL 5.0 GBS pipeline (Glaubitz et al. 2014). Reads were aligned to the *Sorghum bicolor v3.1* reference genome (https://phytozome.jgi.doe.gov/). The TASSEL plugin FSFHap (Swarts et al. 2014), specific for biparental populations, was used for imputing missing genotypes. Imputation was performed independently on each population and chromosome. The “cluster” algorithm was used to infer haplotypes and sites were filtered when the correlation (*r*) with neighboring sites was <0.4 or missing genotype frequency was >0.9 across individuals. All other parameters were maintained at their default values. Following imputation, individual sites with MAF <0.05 were removed. This resulted in 71,856 and 49,617 genome-wide SNPs for the BTx642 and P850029 populations, respectively. The full SNP data sets were used to characterize and compare genomic properties including heterozygosity and LD.

### Recombination bin and genetic map construction

For genetic map construction and subsequent linkage analysis in R/qtl (Broman et al. 2003), RIL genotypes were first converted to ABH allele format where allele “A” is from Parent A, “B” is from Parent B, and “H” is heterozygous. To reduce computational burden and accommodate maximum marker thresholds in the software, SNPs for each RIL population were placed into recombination bins using a method designed by Huang et al. (2009). A 15 SNP sliding window was used to track the Parent1:Parent2 genotype ratio, where Parent1 was the female from the original F_1_ cross. Recombination breakpoints were categorized as either homozygous/homozygous (hom/hom) or homozygous/heterozygous (hom/het). As fully described in Huang et al. (2009), a hom/hom breakpoint was determined when the Parent1:Parent2 ratio of a RIL(s) passed the 8:7 to 7:8 (or vice versa) threshold only once before transitioning into the alternate parent genotype. On the other hand, a hom/het breakpoint was defined when the Parent1:Parent2 ratio passed the same boundary multiple times prior to transitioning into the alternate parent genotype. In cases where sites contained different RILs with a hom/hom and hom/het breakpoint, the site was classified as hom/hom. There were 3178 hom/hom and 1423 hom/het breakpoints for a total of 4601 recombination breakpoints in BTx642. A total of 4154 (3337 hom/hom and 777 hom/het) recombination breakpoints was found in the P850029 population.

Marker sites located between recombination breakpoints were combined into bins. Bin windows were treated as individual markers to construct a genetic map for each population. Genetic distances were estimated from physical positions of each recombination breakpoint using the Kosambi mapping function (Kosambi 1943), with a maximum iteration number of 1000 and the error probability set to 1 × 10^−4^. In R/qtl, the genetic maps for BTx642 and P850029 were converted to cross type “riself”, an abbreviation for “RIL by selfing” (Broman et al. 2003). Because this cross type does not allow for heterozygosity, heterozygous sites across the data set were treated as missing. To take this into account, bin markers with a MAF < 0.05 as a result of both high heterozygosity and residual missing data were eliminated from the genetic map. Finally, markers with severe segregation distortion (*P* < 10^−20^) were removed, resulting in a total of 4589 and 4149 bin markers for BTx642 and P850029, respectively.

### Association and linkage mapping

Genome-wide association studies were performed using the GAPIT package (Lipka et al. 2012) implemented in R. A regular mixed linear model was designated by setting the *group.from* and *group.to* GAPIT parameters equal to the number of individuals in the population, thus each individual genotype was considered a group (Lipka et al. 2012). A kinship matrix was estimated using GAPIT’s default VanRaden method (VanRaden 2008) and incorporated into the model as a random effect. A population structure matrix was not included because kinship adequately accounted for relatedness to control false positives (Fig. S1). Permutation tests as described in Zhang et al. (2015) were previously conducted on the diversity panel SNP data set to determine the association significance cutoff of *P* = 10^−5^ (Boyles et al. 2016.)

R/qtl software (Broman et al. 2003) was used for linkage mapping. The genome-wide LOD significance threshold for BTx642 and P850029 was determined by running *n* = 1000 permutations of the expectation–maximization algorithm. The average LOD score threshold of 1000 permutations with an *α* = 0.05 was LOD = 3.33 for BTx642 and LOD = 3.36 for P850029; therefore, a consistent LOD score of 3.3 was used as the significance threshold for both populations. Interval mapping using the R/qtl scanone() function was used for QTL analysis. This function allowed phenotypic covariates to be incorporated into the model, which was critical to eliminate potential confounding effects on grain quality traits such as grain yield and pericarp color. The same model parameters that were chosen for LOD score threshold determination were used for QTL mapping.

## Results

### Variation of grain quality traits

All grain quality traits displayed normal distributions except amylose, which was bimodal. The GSDP exhibited greater phenotypic variation than the RIL populations in four of the five grain quality traits, with amylose being the lone exception. Lower amylose variation within the GSDP is not surprising given the few known waxy sorghum accessions within the panel. Starch in the GSDP had the greatest range of phenotypic variation (>30%) followed by crude protein, crude fat, and then gross energy (Table 1). Although gross energy in the GSDP contained the smallest amount of phenotypic variation, a 400 kcal kg^−1^ (10.2%) difference was observed between the lowest and highest accession. Between RIL populations, BTx642 had a larger range for crude fat at 4.71% and P850029 had a larger observed range for starch (16.7%), crude protein (8.7%), and gross energy (349 kcal kg^−1^). All grain quality traits were influenced by transgressive segregation, especially starch and crude protein. Mean starch contents from the three parent lines were within 1% of one another; however, both RIL populations contained >15% variation. Transgressive segregants were commonly found in previously studied biparental populations for the three grain macronutrients (Murray et al. 2008) as well as other grain quality traits (Klein et al. 2001).

**Table 1:** Variation in grain quality traits within the grain sorghum diversity panel (GSDP) and two RIL populations

		GSDP		BTx642		P850029	
Trait	Unit	Range	Mean	Range	Mean	Range	Mean
Amylose	% Starch	(0–21.7)	14.2	(0–26.94)	11.49	(0–34.61)	13.26
Starch	% Dry basis	(45.1–75.7)	68	(59.63–74.76)	68.37	(58.55–75.24)	68.54
Crude protein	% Dry basis	(6.95–18.79)	12.47	(8.9–14.98)	11.43	(6.85–15.55)	11.25
Crude fat	% Dry basis	(0.18–5.37)	2.69	(1.25–5.96)	3.07	(0.5–4.86)	2.59
Gross energy	kcal kg^−1^	(3968.3–4371.8)	4140.3	(4036–4350.2)	4183.7	(3965.5–4314.6)	4137.8

**Fig. 1:**
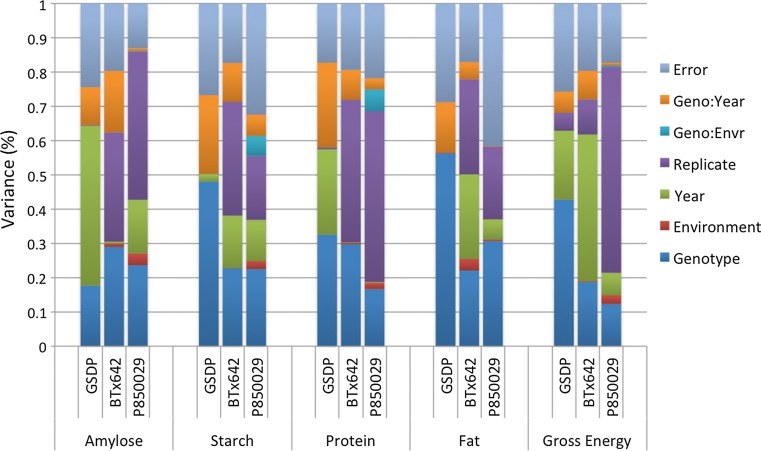
The variance components within each population are presented for amylose, starch, crude protein, crude fat, and gross energy. Percent of genotypic variance across populations and traits ranged from 12.3 to 56.2%. The replicate component was nested within year-environment interaction. *Geno* genotype, *GSDP* grain sorghum diversity panel, *Envr* environment

Estimated genetic variance in the GSDP ranged from 18% (amylose) to above 56% (crude fat). Because the diversity panel was grown in one location over two years, variance due to year accounted for a large percentage of the total phenotypic variation observed (Fig. 1). The one exception was crude fat, which had <1% of phenotypic variance explained by evaluation year. Variance due to genotype in BTx642 and P850029 was less than the estimated genotypic variance in the GSDP, irrespective of trait. Across RIL populations, genotype-by-environment interaction accounted for considerably less of the total variation in crude fat and gross energy content than starch and crude protein. In general, environmental variance alone was very low (<5%) while variance between replicates was surprisingly high. This high replicate variance likely stems from the component being nested within the year-environment interaction term. The residual error in all three populations was similar, averaging approximately 25% (Fig. 1).

### Trait heritability across populations

#### GSDP

Phenotypic correlations (*r*) between years for each grain quality trait ranged from *r* = 0.42 to 0.74. The highest broad-sense heritability (*H* ^2^) among the five traits evaluated was 0.82 for gross energy. Amylose had the lowest broad-sense heritability in the GSDP at 0.56. Heritability across macronutrients was similar (Table 2). Broad-sense heritability for starch (*H* ^2^ = 0.73) was consistent with prior results but calculated heritability estimates for crude protein and fat were both slightly lower than previously observed (Murray et al. 2008).

**Table 2:** Grain sorghum diversity panel correlation coefficients of replicate means for the five major grain quality traits under study and additional agronomic phenotypes

	*H* ^*2* a^	DTA	Height	GNP	TGW	YPP	Amylose	Starch	Crude protein	Crude fat	Gross energy
DTA	0.9	–	0.37***	0.1	−0.19***	0.02	−0.13*	0.08	−0.1	−0.12*	−0.14**
Height	0.94	−0.01	–	−0.02	0.05	0.04	0.09	0	−0.16**	0.19***	0.09
GNP	0.68	0.36***	−0.04	–	−0.12*	0.79***	0.02	0.33***	−0.47***	−0.1*	−0.32***
TGW	0.83	−0.06	−0.07	−0.08	–	0.45***	0	0.22***	−0.16**	−0.21***	−0.68***
YPP	0.68	0.35***	−0.04	0.85***	0.37***	–	0	0.42***	−0.51***	−0.19***	−0.43***
Amylose	0.56	−0.11*	0.06	0	−0.13*	−0.07	–	0.03	0	0.08	0.21***
Starch	0.73	0.03	−0.01	0.33***	0.22***	0.42***	0.01	–	−0.66***	−0.47***	−0.68***
Crude protein	0.65	−0.04	0.01	−0.4***	−0.13*	−0.43***	−0.01	−0.7***	–	0.29***	0.62***
Crude fat	0.72	−0.06	0.03	−0.2***	−0.27***	−0.31***	−0.09	−0.35***	0.22***	–	0.74***
Gross energy	0.82	−0.1	0.03	−0.31***	−0.29***	−0.42***	0.2***	−0.71***	0.6***	0.76***	–

Correlations on the upper right of the diagonal represent 2013 data while 2014 correlations are shown lower left of the diagonal. Agronomic and yield trait data were published previously in Boyles et al. (2016)

*DTA* days to anthesis, *GNP* grain number per primary panicl, *TGW* thousand grain weight, *YPP* grain yield per primary panicle

* Significance at the 0.05 probability level, ** significance at the 0.01 probability level, *** significance at the 0.001 probability level

^a^Broad-sense heritability

#### RILs

Heritability of each trait in BTx642 was relatively similar, ranging from 0.67 (gross energy) to 0.77 (crude fat). Amylose content had the greatest broad-sense heritability in P850029 at 0.86. Starch and crude protein (*H* ^2^ = 0.62) possessed lower heritability in P850029 when compared to both BTx642 and the GSDP. Residual variation was the primary cause of lower heritability for specific grain quality traits (Fig. 1). Primary sources of residual variation include grain processing and trait prediction by NIRS.

### Grain quality trait correlations and relationships with grain yield components

#### GSDP

Because grain macronutrients were measured on a percent dry matter basis, starch, the major macronutrient, expectedly had a strong negative correlation with crude protein and fat (Table 2). Crude protein and fat were positively correlated. Starch content and gross energy also had a strong negative relationship. Meanwhile, crude protein and fat were positively correlated with gross energy. Amylose percentage had a positive correlation with gross energy, meaning low amylose accessions tended to have lower gross energy values. Amylose had no significant correlations with any of the macronutrients in the GSDP.

Three yield component traits were evaluated in the study to examine the phenotypic relationship between grain yield and quality. The three yield components were grain number per primary panicle, 1000-grain weight, and grain yield per primary panicle. Raw data on these yield phenotypes are available in Boyles et al. (2016). In general, yield components had a positive relationship with starch content and negative correlations with crude protein and fat (Table 2). This also resulted in a negative relationship between grain yield traits and gross energy. The positive grain starch—yield relationship was consistent with previous findings (Murray et al. 2008). Amylose was not correlated with yield components, except a slight negative correlation (*r* = −0.15) with 1000-grain weight was detected in 2014. Yield component traits grouped into three levels (low, middle, and high) clearly delineate existing tradeoffs with crude protein and fat as well as gross energy in the GSDP (Table S2).

#### RILs

Trait correlations between the BTx642 and P850029 RIL populations were very similar (Table S3, S4), and grain quality relationships were analogous to those observed in the GSDP. In both RIL populations, amylose had a positive correlation with gross energy, which was consistent with the GSDP. This relationship could be attributed to the tradeoff between amylose and crude fat, which had a strong positive correlation with gross energy. RIL relationships between grain quality and yield traits were similar to GSDP correlations, but several exceptions were identified. In P850029, amylose was negatively correlated with grain number (*r* = −0.27) and yield (*r* = −0.26). There was no tradeoff between 1000-grain weight and amylose in P850029. Also, no yield components were significantly correlated with amylose in BTx642. Another contrast from the GSDP, there was no negative relationship between 1000-grain weight and crude protein in either RIL population. While a negative correlation (*r* = −0.27) between grain weight and crude fat was observed in P850029, no tradeoff was found in BTx642. In fact, there was a slight positive relationship between these traits.

### Genomic characterization of RIL populations

The greater number of polymorphisms and recombination breakpoints in BTx642 (71,856 SNPs; 4601 breakpoints) than the P850029 population (49,617 SNPs; 4154 breakpoints) is indicative of the greater genetic distance of parent BTx642 from BTxARG-1 (data not shown). Of the retained genome-wide SNP sites, there was lower residual heterozygosity in the BTx642 (5%) and P850029 (6.5%) populations than the expected 7% at the F_5_ generation. While the majority of progeny in both populations were largely homozygous across all markers, there were three BTx642 and ten P850029 individuals with >20% of sites heterozygous. One RIL in each population had over 60% of SNP sites carrying an allele from each parent, and these genotypes were removed from downstream analyses given the high likelihood of them being outcrosses. In the BTx642 population, 45.5% of alleles came from parent BTx642 and 49.5% of alleles from parent BTxARG-1. BTxARG-1 was also slightly overrepresented in the P850029 population (47.5 to 46%).

**Fig. 2:**
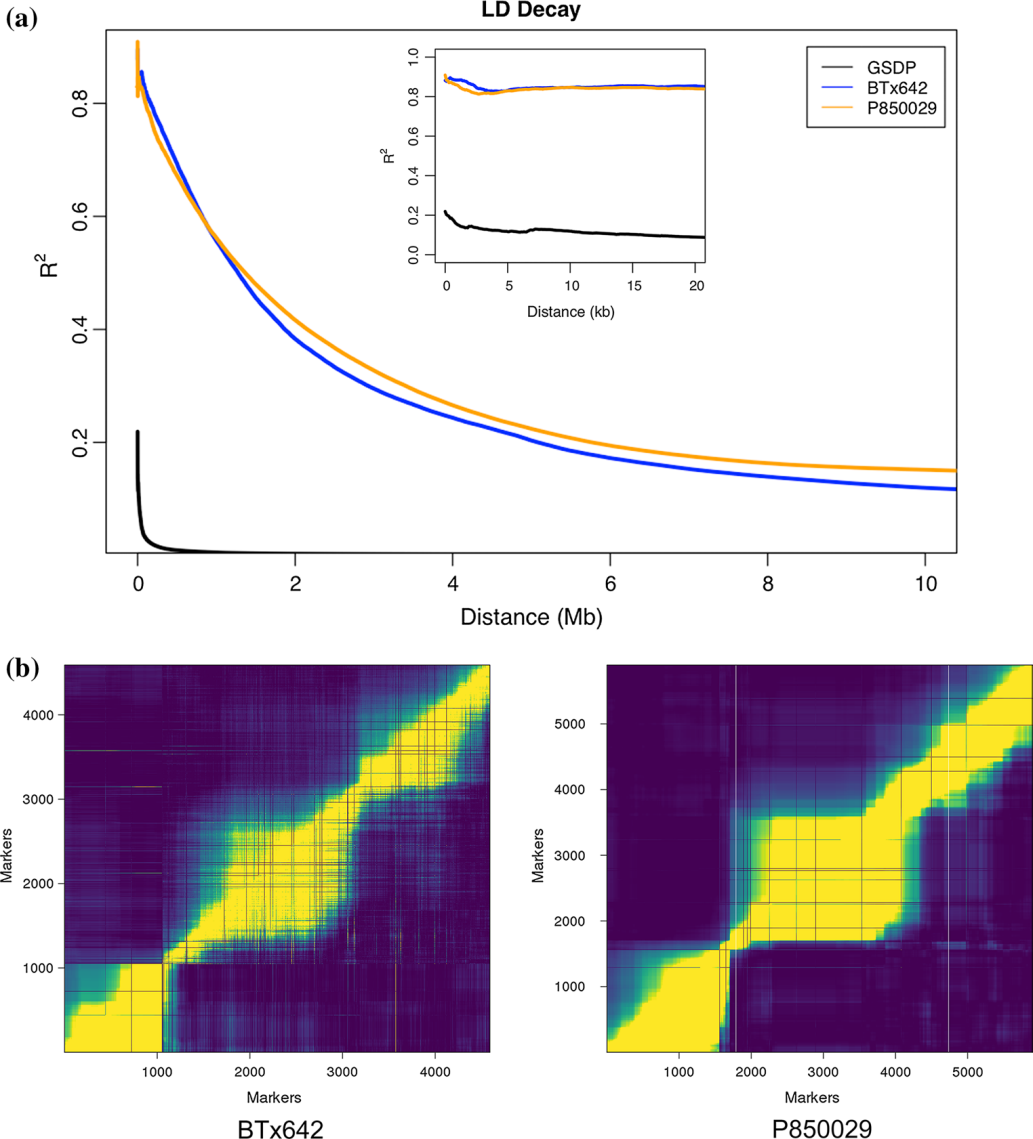
Linkage disequilibrium (LD) decay and recombination fractions of different sorghum populations. **a** Genome-wide average LD (*r* ^*2*^) in the grain sorghum diversity panel (GSDP), RIL population BTx642, and RIL population P850029. Average LD shown is from the mean of all ten sorghum chromosomes. **b** Pairwise recombination fractions in BTx642 and P850029 highlight regional blocks of LD on chromosome 1. The full SNP data set was used to increase marker density. Chromosome-wise recombination fractions for additional chromosomes are shown in Fig S1

The GSDP had a much faster LD decay than both RIL populations (Fig. 2a), as expected. Population P850029 pairwise LD average fell below *r* ^2^ = 0.2 after 5.7 Mb. The BTx642 population reached *r* ^2^ = 0.2 slightly faster at 5.1 Mb. For comparison, average LD decay in the GSDP was *r* ^2^ > 0.2 only when SNPs were physically located within 100 bp of each other. The extent of LD varied both within and across chromosomes for P850029 and BTx642 (Fig. 2b, Fig. S2), which led to a variable mapping resolution that was dependent on QTL position.

Using recombination bins as individual markers, the constructed genetic maps of BTx642 and P850029 had total lengths of 1574.2 and 1416.7 cM, respectively. Average intermarker distance for both populations was ≤0.5 cM for all ten sorghum chromosomes. Segregation distortion, marker deviation from the expected 1:1 Mendelian ratio, was present at various genomic regions in both RIL populations, with P850029 containing more distorted markers than BTx642 (Fig. S3). There were several regions with segregation distortion (*P* < 1e^−10^) in BTx642, one large region on chromosome 1 and other smaller windows on chromosomes 2 and 3. All chromosomes except 1, 3, 8, and 10 contained distorted regions in P850029. Two of these distorted genomic locations in P850029 were near known height loci, *Dw3* and *Dw1*, both of which were segregating in this population. Marker segregation distortion at *Dw3* has been previously identified in a different recombinant inbred population (Murray et al. 2008).

### Genetic mapping consistency within populations

*P* values generated from GWAS results in the GSDP were far less consistent (*r* = 0.11 amylose to *r* = 0.25 gross energy) across years than the mapping reproducibility found in the two RIL populations. In the GSDP, starch associations were positively correlated with crude protein (*r* = 0.34) and gross energy (*r* = 0.32). Crude protein and fat both had a strong positive relationship with gross energy content, which is consistent with QTL mapping results across RIL populations. Genetic Pearson pairwise correlations using LOD scores for each grain quality trait between years and between environments were highly reproducible. In other words, QTL mapping for each trait resulted in relatively consistent LOD scores across the genome regardless of year and environment combination. RIL populations maintained consistency between years, excluding starch in BTx642 and crude fat in P850029 in the SC environment. All grain quality traits had year-to-year genetic correlations above 0.43 in the TX environment with the exception of crude fat in P850029 (*r* = 0.22). Correlations between environments in the same year ranged from 0.15 (BTx642-2015-starch) to 1 (P850029-2014-amylose). LOD scores were more consistent between environments in 2014, with all correlations >0.5 in both RIL populations.

### Identification of grain quality QTLs from association and linkage mapping

#### Amylose

**Fig. 3:**
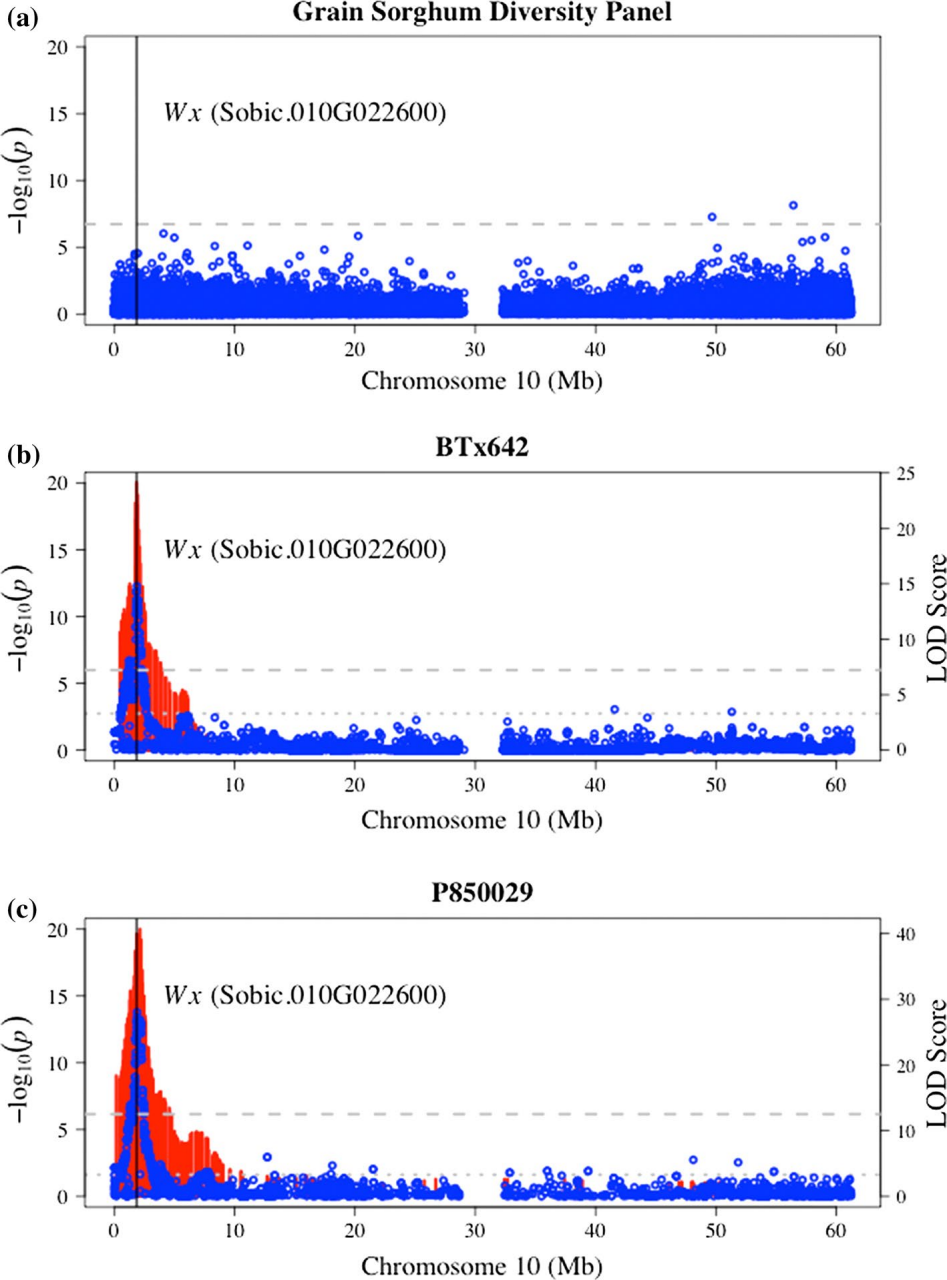
Association mapping of amylose across the grain sorghum diversity panel (GSDP) and the two RIL populations segregating for the waxy trait (low amylose %). **a** As a result of few waxy genotypes in the GSDP and thus a very low minor allele frequency (MAF), no significant associations surrounding the *waxy* (*Wx*) locus (*black vertical line*) are detected. Strong association peaks at the *Wx* locus are detected using phenotypic data from **b** BTx642 and **c** P850029. GAPIT software (Lipka et al. 2012) using the full SNP data set (*blue circles*) and R/qtl software (Broman et al. 2003) using recombination bin markers (*red lines*) both easily identified *Wx* at high resolution. The SNP with highest average significance between the two RIL populations was located 12 kb from *Wx* (*Sobic.010G022600*)

The production of amylose in grain starch is a Mendelian inherited trait (Karper 1933) regulated by the well-characterized *waxy* (*Wx*) gene on chromosome 10 that encodes granule-bound starch synthase I (McIntyre et al. 2008). Because of the very low frequency (4 out of 390 individuals) of waxy sorghum accessions in the GSDP, no strong associations in LD of *Wx* were identified in the amylose GWAS from an obvious lack of statistical power (Fig. 3a). There were several associations from GWAS that surpassed the empirical significance threshold, which could be false positives or possibly additional small effect modifiers of starch composition. The *Wx* locus was, however, easily mapped in both RIL populations at considerably high resolution using both GAPIT and R/qtl software (Fig. 3b, c). The SNP (S10_1877459) with greatest significance between association scans for BTx642 and P850029 was physically located 12 kb from *Wx* (*Sobic.010G022600*). This QTL explained anywhere from 46 to 61% of the genetic variance for amylose, depending on population and environment. Estimated PVE was consistently 42% in P850029 but varied between 30 and 41% in BTx642 (Table S5).

#### Starch

There were only two QTLs and three SNPs in total surpassing the empirical significance cutoff (*P* < 10^−5^) in the starch GWAS. Two of these SNPs at 66.6 Mb on chromosome 2 were separated by 14 bp. The other statistically significant SNP was positioned at 13.8 Mb on chromosome 7. This chromosome 7 QTL explained nearly 10% of the genetic variance for starch in the GSDP.

The strong segregation of yield components in the P850029 biparental population affected association analyses for grain quality traits, including starch content. This was apparent from the significant phenotypic and genetic correlations between grain yield and quality traits (Table S3, S4). Grain number per primary panicle and 1000-grain weight were therefore included into the model as an additive and interactive covariate to account for this strong bias and reduce spurious QTLs. When including both yield components into the model, a QTL on chromosome 5 fell below LOD significance threshold except for crude fat and gross energy in P850029. The QTL for starch, crude protein, and gross energy still co-located with the grain number QTL on chromosome 1. Significant LOD scores across this genomic region were identified in both environments for these three grain quality traits. The peaks for this QTL across grain quality traits in P850029 ranged from 260 to 6.9 Mb, with the starch QTL peak located at 2.3 Mb. The same marker (S1_2294632) had the maximum LOD score for both starch and crude protein in TX15. This SNP was located within a gene encoding a golgi nucleotide sugar transporter (*Sobic.001G029500*), which has been shown to regulate multiple plant development processes in rice (Zhang et al. 2011). Waxy parent BTxARG-1 contained the favorable starch allele while parent P850029 possessed the allele for increased crude protein and gross energy at this multi-trait locus on chromosome 1. Besides the chromosome 1 QTL interval, no additional starch QTLs surpassed the LOD significance threshold in SC15. Another starch QTL in P850029 was mapped near *Ma1* (Murphy et al. 2011), which regulates sorghum flowering time in long days (Murphy et al. 2011). This QTL on chromosome 6 was identified in SC14 and in TX15 (Table 3).

**Table 3:** QTLs that were significant in multiple experiments

Trait	Chromosome	Position (Mb)^a^	SC13^b^	SC14	TX14	SC15^c^	TX15^c^
Amylose	*10*	*1.91*		BP^d^	BP	BP	BP
Starch	*1*	*3.55*		P	P	P	BP
Starch	*2*	*64.91*	G^e^			B	
Starch	*6*	*42.83*		P			P
Starch	*10*	*1.33*		BP	BP		
Starch	10	8.59				B	B
Crude protein	*1*	*4.02*		P	P		BP
Crude protein	1	61.8		BGP			
Crude protein	1	67.73		P			P
Crude protein	*2*	*64.25*		B	B	B	B
Crude protein	*6*	*42.29*		P			P
Crude protein	7	56.53			P		P
Crude protein	9	54.88		P		P	
Crude fat	1	69.88			B	B	
Crude fat	*2*	*63.21*			B	B	
Crude fat	*3*	*1.5*		B		B	
Crude fat	4	14.92		P	P		
Crude fat	*5*	*68.27*	G	GP		P	
Crude fat	6	45.56		P	BP		
Crude fat	*10*	*1.53*		P	BP		B
Crude fat	*10*	*50.12*	G	BG	B	B	B
Gross energy	*1*	*4.43*		P	P	P	P
Gross energy	*3*	*1.99*		P		B	
Gross energy	*5*	*67.12*		GP	P	P	P
Gross energy	*10*	*51.05*	G	BG	BP	B	B

^a^Physical position was calculated using the mean SNP position that had the highest LOD score within the QTL interval from multiple populations and experiments

^b^The grain sorghum diversity panel (GSDP) was the only population evaluated in 2013

^c^The GSDP was not evaluated in 2015

^d^ *B* BTx642 RIL population, *P* P850029 RIL population

^e^ *G* grain sorghum diversity panel

^f^QTL positions that co-located with multiple grain quality traits are in italics

In addition to yield components, pericarp color confounded QTL mapping in BTx642 and thus was introduced into the model as a covariate for starch and other grain quality traits excluding amylose. The P850029 population was not segregating for pericarp color. In BTx642, starch QTLs were identified on chromosomes 1, 2, 3, 4, 6, and 10. The two significant starch SNPs on chromosome 2 from the GSDP GWAS were located within a starch QTL that was identified in SC15. This QTL interval spanned 63.1–68.8 Mb, but the LOD peak was <1 Mb from the significant GWAS SNPs. This locus, along with an additional starch QTL on chromosome 2, co-located with QTLs for crude protein. This observed co-localization was not surprising given the positive genetic correlation between these two traits (Table S3). At both co-localized QTLs on chromosome 2, BTx642 contained the favorable allele for increased starch while the BTxARG-1 allele correlated with increased crude protein. These two QTLs explained more genetic variance for crude protein (12.3 and 20.1%) than starch (9.5 and 8.2%). A minor effect QTL on chromosome 4 was identified in TX15 that was in LD with brittle endosperm1 (*Bt1*), which encodes an ADP-glucose translocator. This enzyme plays a role in the maize starch biosynthesis pathway (Table S1) (Séne et al. 2000).

#### Crude protein

As with starch content, very few SNPs were strongly associated with crude protein variation in the GSDP. In fact, there were no statistically significant associations identified in 2013. In 2014, the protein GWAS in the GSDP identified a genetic variant on chromosome 1 near 60.4 Mb. Two other SNPs associated with crude protein were positioned at 18.8 Mb and 49.6 Mb on chromosome 2. Also on chromosome 2, there was a QTL interval identified in BTx642 from 62.2 to 69.9 Mb. This region was associated with crude protein in the SC and TX environments in both years although the QTL peak in SC14 was ~5 Mb from the peaks observed in 2014. In the SC15 experiment, the chromosome 2 QTL had a peak LOD score of 9.3 and explained 20.1% of the genetic variance. This locus explained 9.9 and 12.2% genetic variance in TX14 and TX15. The strongest QTL for BTx642 mapped in SC14 was nearby at 58.6 Mb on chromosome 2, which was near previously identified protein QTLs (Murray et al. 2008; Rhodes et al. 2016). In TX15, the BTx642 marker with the highest LOD score in the crude protein QTL analysis was on chromosome 1 within a glutamate dehydrogenase gene (*Sobic.001G059100*). Glutamate dehydrogenase plays an important role in N metabolism (Robinson et al. 1991) and maintenance of the C-N balance (Miflin and Habash 2002).

In P850029, there were seven different QTLs for crude protein identified in the SC14 environment alone, with three of these located on chromosome 1. Additional loci were found on chromosomes 2, 3, 6, and 9. The only additional protein QTL found in the TX environment was located on chromosome 7. The crude protein QTL on chromosome 9 detected in SC both years co-located with *Dw1* (Hilley et al. 2016). While protein and height in P850029 had a positive correlation in the SC environment, this protein QTL remained significant when including height into the model as a covariate (Fig. S4). The marker with maximum LOD score within this QTL was fewer than 20 kb from a putative trehalose-6-phosphate synthase transcript, which was highly expressed in sorghum seed tissues during multiple grain filling stages (Davidson et al. 2012). Trehalose-6-phosphate synthase is important in embryo maturation and regulates sugar metabolism within the caryopsis (Eastmond et al. 2002). This QTL near *Dw1* and a protein QTL interval spanning from 260 to 8.6 Mb on chromosome 1 were the most robust, being significant in multiple years and/or environments (Table 3). The chromosome 1 QTL co-located with a protein QTL identified in BTx642 in TX15.

#### Crude fat

**Fig. 4:**
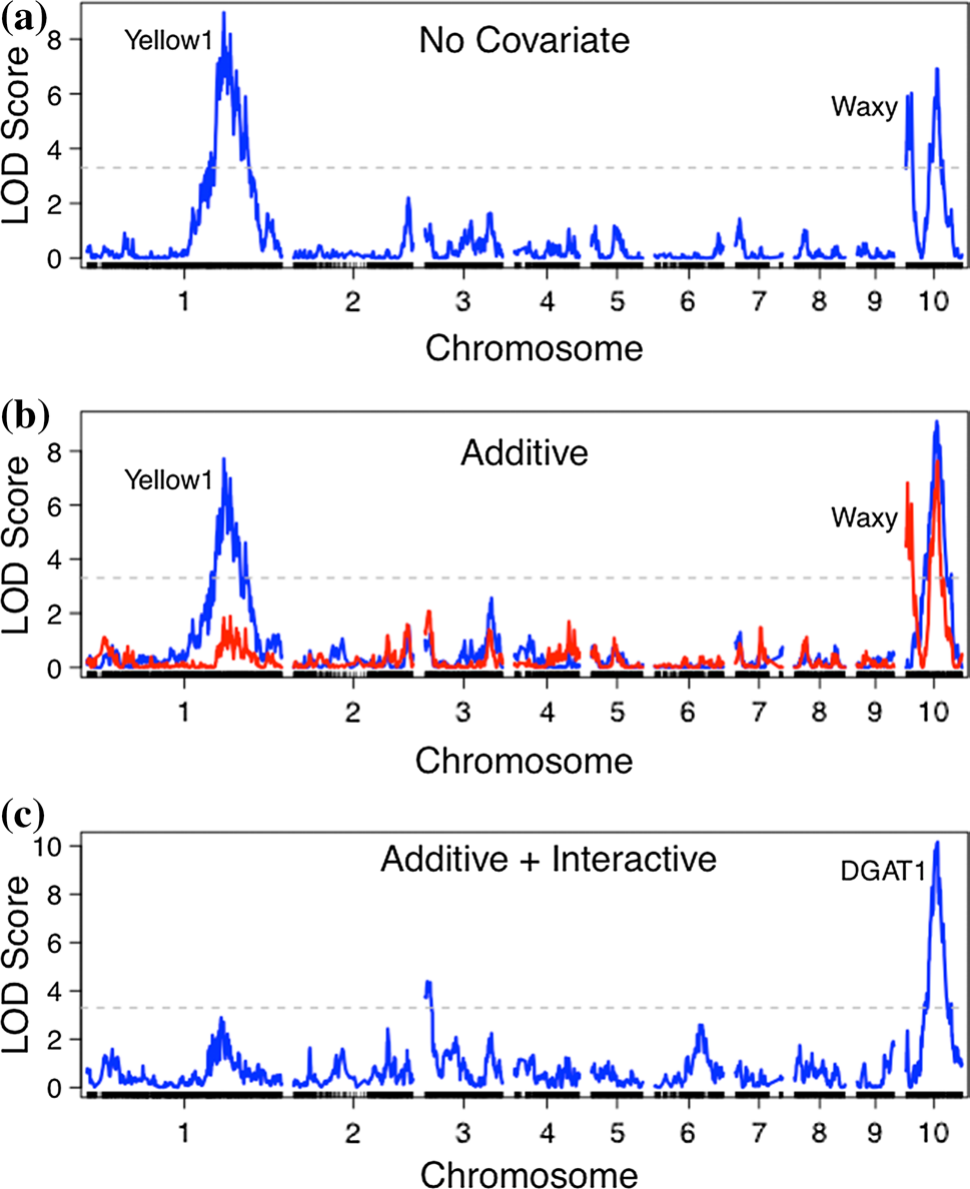
Confounding effects on crude fat QTL mapping in BTx642 were controlled by incorporating trait covariates. **a** QTL mapping results for crude fat with no covariates included. Markers along the *x*-axis are positioned based on genetic distance (cM) **b** Adding pericarp color (*red*) and amylose (*blue*) as additive covariates eliminated false-positive QTLs at the *yellow1* and *waxy* loci, respectively. **c** Including amylose as an additive covariate and pericarp color as an interactive covariate eliminated false-positive QTLs and increased the peak LOD score found at 50 Mb on chromosome 10 near the homologue of maize *diacylglyceroal O*-*acyltransferase 1* (*DGAT1*)

**Fig. 5:**
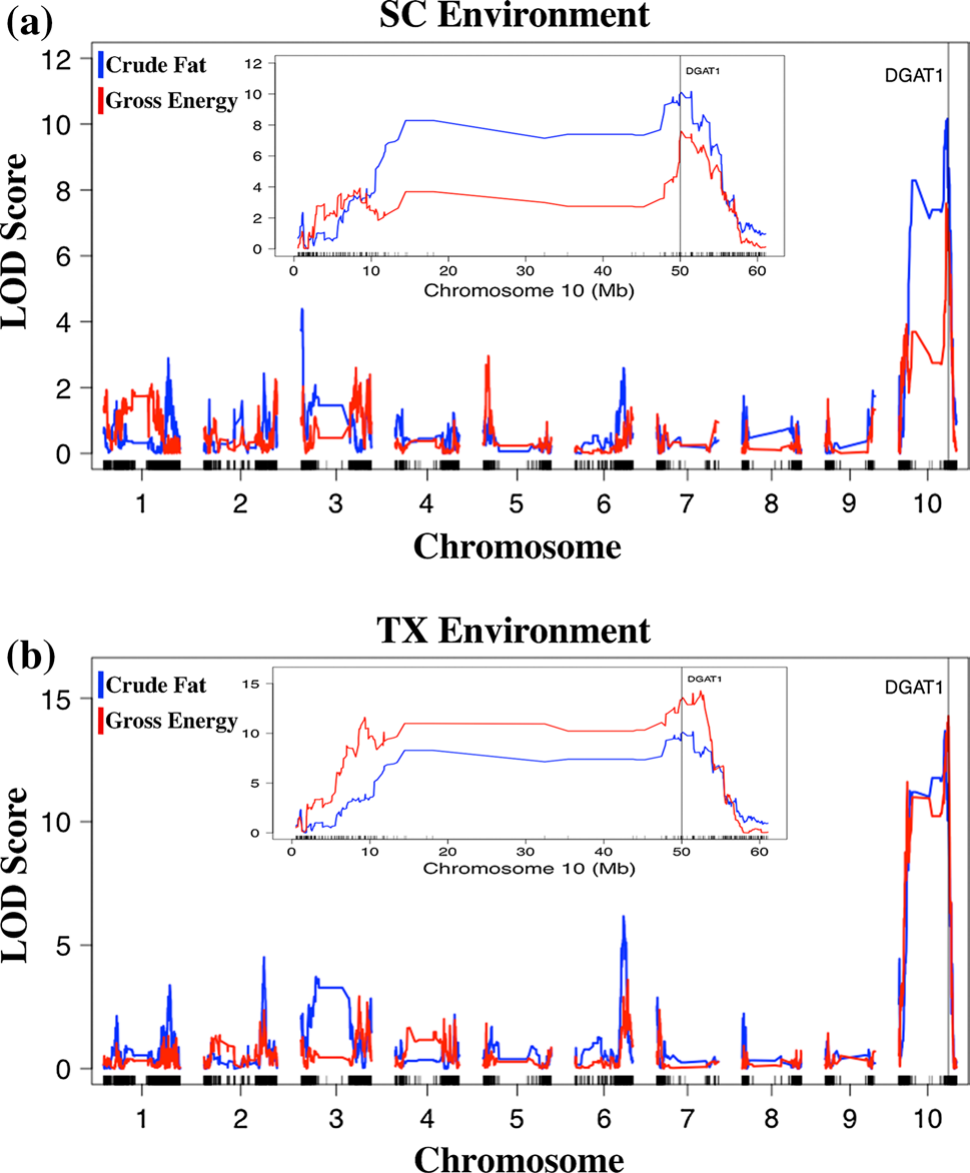
Genome-wise LOD scores for crude fat and gross energy in the 2014. **a** SC and **b** TX environments. Inserts for each environment highlight the major QTL on chromosome 10 that encompasses the *diacylglyceroal O*-*acyltransferase 1* (*DGAT1*) locus. Markers are distributed along chromosomes based on physical position in megabases (Mb)

Initial QTL analysis for crude fat in BTx642 revealed that the trait was confounded by pericarp color and amylose content (Fig. 4a, b). To account for this relationship, amylose was incorporated as an additive covariate and pericarp color as an interactive covariate. This covariate model identified a strong QTL for crude fat located on chromosome 10 (Fig. 4c). The QTL was identified in the GSDP and the BTx642 biparental population. In the GSDP, four SNPs in tight linkage at 50 Mb on chromosome 10 were significantly associated with crude fat content in both 2013 and 2014. In fact, these SNPs were all ranked in the top five each year based on *P* values generated from the GWAS mixed linear model. SNPs with peak LOD scores in BTx642 were located between 49.1 and 51.5 Mb although there was a larger confidence interval spanning across chromosome 10 that enveloped the centromere (Fig. 5). This QTL explained 28.1% of the genetic variance for 2014 crude fat content in TX and 21.7% in SC. In TX15, the chromosome 10 QTL had nearly identical significance, explaining 27.6% genetic variance. The next QTL (LOD = 6.2) of largest significance was found in the TX14 environment and had an estimated 10.6% PVE, which was located at 50.9 Mb on chromosome 6. A third crude fat QTL of similar significance was identified at 1.5 Mb on chromosome 3 in both years in SC.

The P850029 RIL population contained several significant QTLs across the genome for crude fat, although none with near the effect of the chromosome 10 QTL identified in BTx642. There was a consistent QTL found across environments on chromosome 4 at 14.9 Mb (Table 3), and this locus explained 8 and 5.4% of the genetic variance for crude fat in SC14 and TX14, respectively. Other minor but significant crude fat QTLs were located on chromosomes 1, 2, 5, and 6. These QTLs, however, were environment-specific.

#### Gross energy

Multiple gross energy QTLs identified by association and interval mapping co-located with QTLs for crude protein and fat. This finding is consistent with the positive phenotypic relationship of gross energy with these two macronutrients. There were eight physically unlinked (>20 kb) SNPs significantly associated with gross energy in the GSDP. The top ranked SNP (smallest *P* value) associated with crude fat in 2013 and 2014 represented two of these eight SNPs, and no significant gross energy SNP was ranked below 516 in the crude fat GWAS results out of 286,896 total markers. Significant SNPs were scattered across five different chromosomes: 1, 2, 5, 6, and 10. The gross energy GSDP associations and RIL linkage analysis consistently identified the 49-51 Mb region on chromosome 10 as a major QTL. Including the crude fat phenotype as a covariate did not reduce the LOD score of the chromosome 10 QTL in BTx642 to confirm against the possibility of a confounding effect. This locus contributed significantly more to crude fat variation in the TX environment, explaining 29.1 and 24.5% of the total genetic variance in 2014 and 2015, respectively. Aside from the chromosome 10 locus, a SNP (S5_65131230) located at 65.1 Mb on chromosome 5 was strongly associated with gross energy content in 2013 (Rank: 15) and 2014 (Rank: 6) in the GSDP. This SNP fell within a gross energy QTL that was identified in both environments and years in the P850029 population. The peak of this QTL, which explained 14.6 and 11.7% of the genetic variance in SC14 and TX14, respectively, was 500 kb from a cluster of kafirin genes previously identified using comparative genomics (Xu and Messing 2008; Wu et al. 2013).

In total, there were seven non-overlapping QTLs of significance in BTx642 and eight QTLs in P850029. Five of these 15 QTLs were identified in multiple experiments (Table 3). The P850029 gross energy QTL on chromosome 5 was identified in each of the four experiments along with the chromosome 10 QTL in BTx642. In addition, the P850029 multi-trait QTL on chromosome 1 also co-located with a gross energy QTL that was significant in all experiments except SC15. The multi-trait locus included starch and crude protein but, interestingly, did not co-locate with crude fat like the QTLs on chromosomes 5 and 10.

### Grain quality QTL and allele effects on grain yield components

Inspection of each significant crude protein QTL revealed the majority of alleles for increased protein appeared to lower grain yield, supporting the phenotypic tradeoff between these two traits within the GSDP (Boyles et al. 2016) and RIL populations. Only five of the 25 significant QTLs across both RIL populations contained a favorable allele for protein without causing an apparent decrease in grain yield. Two of these QTLs were identified on chromosome 2 in the BTx642 population, with one peak at 58.6 Mb and another at 68.3 Mb, but the QTL intervals did not overlap. The other three protein QTLs were found in P850029 on chromosomes 6, 8, and 10. Parent BTxARG-1 contained the allele for increased protein at all three loci while the BTx642 allele increased crude protein at the two QTLs on chromosome 2. The three P850029 protein QTLs each contributed small effects (<5% PVE), but the BTx642 QTL at 58.6 Mb on chromosome 2 explained 9.4% of the phenotypic variance. Additional accessions within the GSDP that contained this favorable allele were primarily milo types as well as three broomcorn sorghums. This locus co-located with a protein QTL previously mapped in another biparental population (Murray et al. 2008) as well as in diverse germplasm (Rhodes et al. 2016).

An average 1000-grain weight of 21 g and grain yield per panicle of 29.3 g were both slightly lower among lines carrying the high crude fat allele (S10_50089573) than the respective grand mean of the entire GSDP (Boyles et al. 2016). There were several genotypes, however, containing the favorable allele with high yield component traits, including the top grain-yielding line (Standard Early Hegari). This suggests incorporating this allele into additional elite germplasm to increase crude fat and thus gross energy content will not impose an adverse effect on grain yield. Conversely, selecting for the predominant allele to lower crude fat content in the grain would potentially allow for more percent starch desired for biofuel conversion.

At the gross energy locus located at 65.1 Mb on chromosome 5, there were 22 and 12 accessions in the GSDP carrying one and two copies of the favorable ‘T’ allele, respectively. Both ‘AT’ and ‘TT’ genotypes increased gross energy content by nearly 3%, thus displaying dominance over the major allele. Interestingly, while accessions heterozygous at this locus had a 3 g lower grain yield per primary panicle on average than homozygous ‘AA’ genotypes, the 12 homozygous ‘TT’ accessions did not possess lower grain yields. Introgression of the favorable minor allele from one of these 12 accessions into elite grain lines could result in high yielding sorghums with increased digestible energy for the feed industry.

## Discussion

### Assessment of mapping strategies

**Fig. 6:**
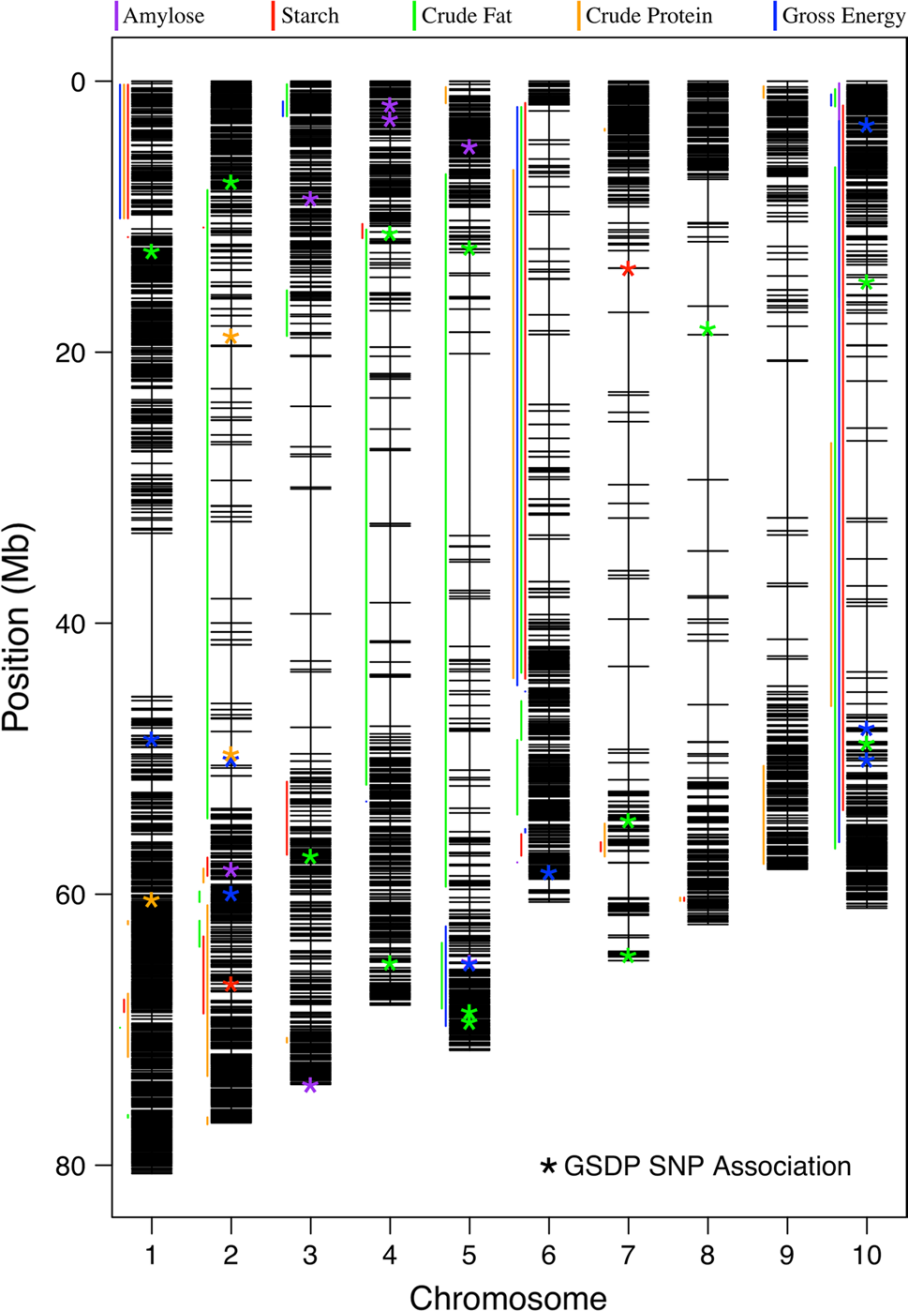
Physical map highlighting the positions of significant SNP associations and QTL intervals identified throughout the study for five grain quality traits. Genome-wide association studies using data from the grain sorghum diversity panel (GSDP) generated SNP associations and two segregating RIL populations were studied to map QTLs. *Asterisks* denote SNP associations and *vertical lines* correspond to locations of QTL intervals. Specific locations for all QTLs are listed in Table S5, along with corresponding information

Inability of GWAS to detect many consistent grain quality QTLs in the GSDP can likely be attributed to low allele frequency, genetic background effects, and lack of statistical power (Korte and Farlow 2013). Lack of consistent results from GWAS is reiterated by lower year-to-year pairwise SNP correlations when compared to LOD score correlations found in the two RIL populations. Additionally, the amylose trait epitomizes how rare alleles in diverse association panels go undetected. Previous research on sorghum (Rami et al. 1998; de Alencar Figueirido et al. 2010) and in other crops has shown that grain macronutrients are quantitative traits, regulated by multiple genes. Small effect genetic variants influencing grain quality traits within the GSDP were unable to be elucidated in this study, especially across environments. However, there were larger effect QTLs that were consistently identified across environments in both years and, in some instances, in multiple traits. There were also grain quality QTLs found in one or both RIL populations that encompassed significant SNPs identified in GWAS (Table 3; Fig. 6). When this overlap occurred, SNPs identified in GWAS were physically located closer to the predicted candidate gene than the corresponding QTL peak although it remains speculative whether the candidate gene is in fact causative. Nevertheless, these robust genomic regions provide excellent targets for molecular breeders to manipulate grain composition in a non-GMO crop and adapt sorghum genotypes to meet the needs of diverse grain markets.

Amylose content was included in this multi-population study for proof of concept. The *Wx* locus located on chromosome 10 was not detected with association analysis in the GSDP, but was fine-mapped in both RIL populations. The allele frequency at this locus in the GSDP was 0.01 with only four waxy sorghums included in the panel (BTxARG-1, BTx615, RTx2907, and Standard White Milo). This low MAF allows *Wx* to go undetected in GWAS. While the average LD was quite large in the biparental populations, certainly much greater than in the GSDP, the amylose QTL peak was located just 12 kb from the *Wx* locus (*Sobic.010G022600*). This finding suggests that merging high-throughput genotyping with segregating populations can potentially narrow down QTL intervals, benefitting future gene discovery.

### Genetic targets and candidate genes for grain quality improvement

Based on historic US accessions including BOK11, Combine Kafir-60, Dwarf Yellow Milo, and Wheatland—all of which have high starch content—development and utilization of high grain starch sorghums occurred long ago. The positive relationship between grain yield and grain starch is likely the underlying root cause of why high starch lines are so widely used in the sorghum breeding pipeline. As grain sorghum selections focused on plants with large panicles and high grain set, starch content concurrently increased over time in breeding populations. However, there were a few starch loci identified where favorable alleles were much more prominent in non-elite accessions. The minor allele at a starch QTL at 58.6 Mb on chromosome 2 that was identified in the P850029 population had a negative effect on starch content. This starch QTL co-located with crude protein. Among the accessions containing the unfavorable allele at this locus were most elite grain sorghums such as Caprock, Combine 7078, Dwarf Yellow Milo, Plainsman, and Wheatland. A second QTL, located on chromosome 10, contained a minor allele that was associated with increased starch content. There were 135 accessions in the GSDP with the favorable starch variant, with a mixture of elite and exotic sorghums carrying the allele.

While most high starch genotypes in the GSDP possessed the majority of favorable alleles at identified QTLs, no one accession possessed all of the beneficial variants across the 12 significant starch loci. Each of the accessions PI34911 and PI656031 contained 11 favorable alleles. PI34911 (F.C.I. 4201) had the fourth highest starch content (72.6%) while PI656031 (CE151-262-A1) had an average starch content of 72%, which was ninth highest in the GSDP. Introgression of additional favorable starch alleles into elite breeding lines may further increase percent starch in the grain, which would be desirable for ethanol conversion (Wu et al. 2007; Wang et al. 2008) and other starch-based sorghum products. Based on allelic variation in the GSDP, gene action among the 12 starch loci was a combination of dominant and additive. Several QTLs displayed underdominance in which heterozygotes had a lower mean starch than homozygous accessions although this observation is subject to selection bias as heterozygous lines were underrepresented within the GSDP.

While the grain quality GWAS failed to detect many significant associations, one association with crude protein was located <10 kb from a putative gene (*Sobic.001G315800*) encoding quinate dehydrogenase. This enzyme is responsible for one of the seven steps in the shikimate pathway, the pathway responsible for synthesis of the aromatic amino acids phenylalanine, tryptophan, and tyrosine (Weaver and Herrmann 1997). Additionally, one of the two significant SNPs on chromosome 2 significantly associated with protein was in LD with *Sobic.002G160600* that encodes an indole-3-glycerol phosphate synthase, which catalyzes an important reaction in tryptophan biosynthesis (Tzin and Galili 2010). The chromosome 1 SNP located near *Sobic.001G315800* had a high MAF of 0.48 while the SNP association near putative indole-3-glycerol phosphate synthase was just above the MAF cutoff (0.06). This rare minor allele had a positive effect on crude protein content, based on results generated from GAPIT. Elite breeding line Tx430 and waxy sorghum Tx2907, both, contained this favorable allele at 18.8 Mb on chromosome 2. At 15.2%, Tx2907 had the seventh highest average protein content between years in the GSDP. Accessions heterozygous at this locus (*n* = 32) possessed low grain protein to suggest dominance for decreased protein.

Association and QTL mapping identified a QTL associated with crude fat content, which was consistently found in two different environments and years. This QTL located on chromosome 10 also associated strongly with gross energy content. Given the positive relationship between crude fat and gross energy, this finding is not surprising. This QTL, which explained up to 9.7% of the crude fat phenotypic variation in the GSDP and 21.5% in the BTx642 RIL population, encompassed the *diacylglycerol O*-*acyltransferase 1* (*DGAT1*) maize homologue (*Sobic.010G170000*). The QTL peak in TX15 was positioned within the *DGAT1* transcript. DGAT is responsible for the final enzymatic and rate-limiting step in the maize lipid biosynthesis pathway (Zheng et al. 2008). A single amino acid insertion within maize *DGAT1* was shown to have a strong effect on lipid content and composition (Zheng et al. 2008). In sorghum, *Sobic.010G170000* is predominantly expressed in the developing embryo in genotype BTx623 (Davidson et al. 2012). Alleles at this locus were not segregating in the P850029 population; however, there were 57 genotypes within the GSDP carrying the favorable allele at this QTL. These accessions consisted of both elite and exotic lines and comprised all five major sorghum botanical races based on classifications from the USDA Germplasm Resources Information Network and Casa et al. (2008). Although digestible energy was not directly evaluated, crude fat is energy dense and gross energy has a strong correlation with digestible energy in wheat and barley (Bhatty and Wu 1974). Thus, this crude fat QTL and the gross energy locus identified on chromosome 5 are targets to potentially increase the energy value of grain sorghum. The latter QTL was located near a large cluster of kafirin-related genes at 67.6 Mb. These protein bodies can reduce protein and overall digestibility in sorghum (Oria et al. 2000; Duodu et al. 2003). This chromosome 5 QTL could be pleiotropic, given it co-located with the most significant 1000-grain weight QTL in P850029.

### Grain yield and quality tradeoffs

Trait correlations in the GSDP between previously published grain yield components (Boyles et al. 2016) and grain quality traits under study suggest increasing crude protein and fat will lower grain yield (Table 2). This correlation was reiterated with phenotypic data collected in both RIL populations (Tables S3, S4). Based on trait relationships, the grain yield–protein tradeoff primarily arises from a decrease in grain number while increasing grain fat content caused a greater reduction in 1000-grain weight in the GSDP (Table S2). This relationship between crude fat and 1000-grain weight may be influenced by the size ratio of embryo and endosperm in the caryopsis (Zheng et al. 2008). The strong negative relationship that grain yield has with protein content has been observed repeatedly across cereal grains (Slafer et al. 1990; Simmonds 1995; Feil 1997). Because these two macronutrients have roughly equal (protein) or higher (fat) caloric value than starch, a tradeoff between grain yield and gross energy is observed (USDA-NAL-Food and Nutrition Information Center 2016). This tradeoff is disadvantageous to the animal feed industry, which is in search of grains that have higher feed efficiency. Feed rations including grain from high-oil maize cultivars resulted in increased weight gain in cattle, poultry, and swine (Lambert et al. 2004). Thus, identified crude protein and fat QTLs that appear to be exceptions and do not reduce yield were highlighted previously in the results.

On the other hand, the positive starch—grain yield correlation makes breeding for high starch grain amenable to ethanol fermentation a feasible task. Furthermore, no tradeoff was observed between amylose and grain yield in the GSDP and BTx642 population although this evaluation of inbred lines conflicts with yield results from waxy and non-waxy sorghum hybrids (Rooney et al. 2005). On average, waxy RILs in the P850029 population actually yielded more grains per panicle than non-waxy lines in 2015. The ability to develop high starch, low amylose (waxy) genotypes would increase ethanol conversion efficiency in sorghum (Wu et al. 2007; Yan et al. 2011). The monogenic waxy trait could be easily introgressed once elite, high starch genotypes are developed. This breeding target is important given the demand for ethanol has increased sharply as a result of the incorporation of this renewable fuel into gasoline blends (Wang et al. 2008).

## Conclusions

Grain quality improvement in cereal crops continues to be an important area of research as cereals represent the largest constituent of global food supplies (Gilland 2002). The combination of association and linkage mapping across environments identified both robust genomic regions that affect grain quality in different genetic backgrounds and also environment-specific QTLs for the SC Coastal Plain and Central TX. In some instances, high-density SNP markers provided high resolution linkage mapping as shown for the amylose QTL peak located 12 kb from the waxy locus (Fig. 4b, c). Furthermore, several markers with maximum LOD scores for grain quality traits were located within transcripts of candidate genes including a glutamate dehydrogenase candidate for protein and the crude fat *DGAT1* gene. However, there were also large QTL intervals identified across multiple environments with QTL peaks separated by several Mb, suggesting resolution is likely dependent on regional LD in the population. While this study corroborates previous findings (Simmonds 1995) of a tradeoff between grain quality (high crude protein, crude fat, and gross energy) and grain yield, favorable alleles for quality traits were also identified that exhibit no adverse impact on yield components. These exceptions provide a means to develop genotypes with higher-quality grains for the food and feed industries that will still be productive for the farmer. Incorporation of genetic markers within these beneficial QTLs into marker-assisted and genomic selection pipelines will be useful for grain quality improvement in sorghum and potentially additional cereal crops.

### Author contribution statement

REB, BKP, SK, and WLR conceived the study. REB and BKP designed the experiments. BKP and WLR provided seed for the recombinant inbred study. REB, BKP, BLR, KJZ, MTM, and ZB collected field data. REB and EAC developed genetic data sets and carried out association and linkage mapping. REB wrote the manuscript. All authors read and approved the manuscript.

## Electronic supplementary material

Below is the link to the electronic supplementary material.

Supplementary material 1 (DOCX 1826 kb)

Supplementary material 2 (XLSX 61 kb)
