# Supplementary material for: Genetic dissection of sorghum grain quality traits using diverse and segregating populations
Source: Theor Appl Genet. 2016 Dec 27;130(4):697–716. doi: 10.1007/s00122-016-2844-6 (PMC5360839; doi:10.1007/s00122-016-2844-6)
Supplement: Supplementary file 1 — Supplementary material 1 (DOCX 1826 kb) [file 122_2016_2844_MOESM1_ESM.docx]

**Supplemental Material**

**Tables**

| **Table S1.** Genome location and sequence similarity of sorghum homologues of known genes in the maize starch biosynthesis pathway. | | | | | |
| --- | --- | --- | --- | --- | --- |
| Gene | Maize | Sorghum | Chromosome | Position (Mb) | Similarity (%) |
| sucrose synthase1 (*Sus1*) | GRMZM2G152908 | Sobic.001G344500 | 1 | 63321126 - 63327577 | 99.6 |
| amylose extender1 (*Ae1*) | GRMZM2G032628 | Sobic.004G163700 | 4 | 51292092 - 51304326 | 97.4 |
| waxy (*Wx*) | GRMZM2G024993 | Sobic.010G022600 | 10 | 1860965 - 1865278 | 96.1 |
| brittle endosperm2 (*Bt2*) | GRMZM2G068506 | Sobic.007G101500 | 7 | 25707997 - 25712978 | 96.9 |
| sugary1 (*Su1*) | GRMZM2G138060 | Sobic.007G204600 | 7 | 63511383 - 63518338 | 94.3 |
| shrunken1 (*Sh1*) | GRMZM2G089713 | Sobic.010G072300 | 10 | 5859074 - 5867276 | 98.9 |
| brittle endosperm1 (*Bt1*) | GRMZM2G144081 | Sobic.004G085100 | 4 | 7084757 - 7086819 | 85.1 |
| UDP-glucose pyrophosphorylase (*Ugp1*) | GRMZM2G032003 | Sobic.002G291200 | 2 | 66932744 - 66940278 | 97.5 |
| shrunken2 (*Sh2*) | GRMZM2G429899 | Sobic.003G230500 | 3 | 57001841 - 57007815 | 95 |

| Table S2. Grain quality trait means in the Grain Sorghum Diversity Panel (GSDP) across yield component categories. As yield increases, starch increases while crude protein, crude fat, and gross energy decrease. | | | | | | |
| --- | --- | --- | --- | --- | --- | --- |
|  | 1000-Grain Weight | | | Grain Yield per Primary Panicle | | |
|  | Low | Middle | High | Low | Middle | High |
| Limits | < 20 g | 20 g ≤ *x* ≤ 25 g | > 25 g | < 30 g | 30 g ≤ *x* ≤ 40 g | > 40 g |
| *n* | 113 | 145 | 127 | 157 | 116 | 112 |
| Starch | 67.21 | 68.57 | 68.71 | 67.14 | 68.6 | 69.31 |
| Crude Protein | 12.79 | 12.19 | 12.23 | 12.99 | 12.24 | 11.68 |
| Crude Fat | 2.95 | 2.62 | 2.54 | 2.87 | 2.59 | 2.54 |
| Gross Energy | 1890.2 | 1875.1 | 1871.7 | 1889.4 | 1873.7 | 1868.1 |

| **Table S3.** Phenotypic (upper right of diagonal) and genetic (lower left of diagonal) correlations from the BTx642 RIL population in 2014. | | | | | | | | | | | | | | | | | |
| --- | --- | --- | --- | --- | --- | --- | --- | --- | --- | --- | --- | --- | --- | --- | --- | --- | --- |
|  | SC Peri | SC DTA | TX DTA | TX Hgt | SC GNP | SC TGW | SC YPP | SC Amy | TX Amy | SC Star | TX Star | SC Prot | TX Prot | SC Fat | TX Fat | SC GE | TX GE |
| SC Peri | - | NS | NS | NS | NS | NS | NS | NS | NS | 0.47 | 0.46 | -0.29 | -0.23 | -0.45 | -0.4 | -0.44 | NS |
| SC DTA | -0.2 | - | 0.4 | NS | 0.24 | -0.23 | NS | NS | NS | NS | NS | NS | NS | 0.21 | NS | NS | NS |
| TX DTA | 0.06 | 0.56 | - | NS | NS | -0.19 | NS | NS | NS | NS | -0.2 | NS | 0.2 | NS | NS | NS | 0.22 |
| TX Hgt | -0.11 | -0.06 | NS | - | NS | NS | NS | NS | NS | NS | NS | NS | NS | NS | NS | NS | NS |
| SC GNP | -0.15 | 0.29 | 0.18 | 0.1 | - | -0.37 | 0.89 | NS | NS | 0.41 | 0.21 | -0.52 | -0.27 | NS | NS | -0.32 | NS |
| SC TGW | -0.14 | 0.2 | NS | 0.11 | 0.36 | - | NS | NS | -0.19 | NS | NS | NS | NS | NS | NS | NS | NS |
| SC YPP | 0.08 | NS | 0.09 | 0.08 | 0.64 | 0.05 | - | NS | -0.19 | 0.48 | 0.23 | -0.54 | -0.24 | -0.23 | -0.19 | -0.41 | NS |
| SC Amy | 0.05 | -0.07 | -0.09 | -0.06 | -0.11 | -0.15 | NS | - | 0.83 | -0.45 | -0.44 | NS | NS | 0.57 | 0.48 | NS | NS |
| TX Amy | -0.06 | -0.05 | -0.08 | -0.05 | -0.06 | -0.09 | 0.05 | 0.98 | - | -0.5 | -0.46 | 0.28 | NS | 0.45 | 0.48 | NS | NS |
| SC Star | NS | 0.11 | -0.06 | NS | NS | 0.15 | 0.16 | 0.6 | 0.62 | - | 0.69 | -0.77 | -0.52 | -0.67 | -0.47 | -0.65 | -0.29 |
| TX Star | -0.08 | 0.06 | NS | -0.06 | NS | NS | 0.11 | 0.46 | 0.49 | 0.6 | - | -0.52 | -0.76 | -0.55 | -0.65 | -0.49 | -0.63 |
| SC Prot | 0.12 | 0.11 | NS | 0.13 | 0.19 | 0.53 | 0.1 | -0.06 | NS | 0.32 | 0.05 | - | 0.6 | 0.24 | 0.19 | 0.5 | 0.19 |
| TX Prot | -0.05 | 0.23 | 0.09 | NS | 0.35 | 0.31 | 0.18 | -0.09 | -0.06 | NS | 0.23 | 0.56 | - | 0.24 | 0.21 | 0.35 | 0.48 |
| SC Fat | 0.11 | 0.12 | 0.13 | -0.12 | -0.05 | NS | -0.1 | NS | NS | 0.2 | 0.46 | NS | 0.08 | - | 0.73 | 0.67 | 0.43 |
| TX Fat | 0.07 | 0.12 | 0.13 | -0.08 | -0.07 | 0.12 | -0.09 | NS | NS | 0.23 | 0.54 | 0.07 | 0.13 | 0.84 | - | 0.52 | 0.65 |
| SC GE | -0.22 | 0.16 | 0.05 | NS | -0.06 | -0.11 | -0.07 | NS | NS | 0.16 | 0.43 | -0.12 | 0.05 | 0.62 | 0.55 | - | 0.56 |
| TX GE | -0.09 | 0.24 | 0.19 | -0.08 | -0.09 | NS | -0.09 | 0.08 | 0.08 | 0.28 | 0.57 | NS | 0.14 | 0.79 | 0.84 | 0.72 | - |
| ^a^ SC, South Carolina environment; TX, Texas environment | | | | | | | | | | | | | | | | | |
| ^b^ Peri, pericarp color; DTA, days to anthesis; Hgt, plant height; GNP, grain number per primary panicle; TGW, 1000-grain weight; YPP, grain yield per primary panicle; Amy, amylose; Star, starch; Prot, protein; GE, gross energy | | | | | | | | | | | | | | | | | |
| ^c^ NS, no significance at the *p* = 0.01 probability level | | | | | | | | | | | | | | | | | |

| **Table S4.** Phenotypic (upper right of diagonal) and genetic (lower left of diagonal) correlations from the P850029 RIL population in 2014. | | | | | | | | | | | | | | | | | |
| --- | --- | --- | --- | --- | --- | --- | --- | --- | --- | --- | --- | --- | --- | --- | --- | --- | --- |
|  | SC DTA | TX DTA | SC Hgt | TX Hgt | SC GNP | SC TGW | SC YPP | SC Amy | TX Amy | SC Star | TX Star | SC Prot | TX Prot | SC Fat | TX Fat | SC GE | TX GE |
| SC DTA | - | 0.49 | 0.27 | 0.21 | 0.24 | NS | 0.29 | NS | NS | NS | NS | NS | NS | NS | NS | NS | NS |
| TX DTA | 0.34 | - | NS | NS | NS | NS | NS | NS | NS | NS | NS | NS | NS | NS | NS | NS | 0.19 |
| SC Hgt | NS | -0.06 | - | 0.86 | NS | 0.35 | 0.37 | NS | NS | NS | NS | NS | 0.22 | -0.17 | -0.24 | -0.21 | NS |
| TX Hgt | -0.06 | -0.07 | 0.88 | - | 0.16 | 0.31 | 0.38 | NS | NS | NS | NS | NS | 0.18 | NS | -0.22 | -0.19 | NS |
| SC GNP | 0.11 | 0.17 | -0.1 | -0.06 | - | -0.38 | 0.76 | NS | NS | 0.23 | 0.22 | -0.54 | -0.35 | NS | NS | NS | NS |
| SC TGW | 0.08 | 0.25 | -0.05 | -0.05 | 0.51 | - | 0.3 | 0.19 | 0.19 | 0.26 | NS | NS | 0.25 | -0.5 | -0.44 | -0.46 | -0.26 |
| SC YPP | NS | NS | NS | 0.1 | 0.66 | 0.23 | - | NS | NS | 0.44 | 0.27 | -0.49 | -0.19 | -0.35 | -0.28 | -0.46 | -0.32 |
| SC Amy | -0.05 | -0.09 | -0.08 | -0.08 | NS | NS | -0.12 | - | 0.89 | 0.37 | 0.28 | NS | NS | -0.46 | -0.44 | NS | NS |
| TX Amy | NS | -0.09 | -0.08 | -0.09 | NS | NS | -0.12 | 1 | - | 0.4 | 0.3 | NS | NS | -0.49 | -0.52 | -0.16 | NS |
| SC Star | NS | 0.05 | 0.16 | 0.06 | 0.43 | 0.23 | 0.15 | 0.54 | 0.53 | - | 0.62 | -0.68 | -0.39 | -0.74 | -0.52 | -0.77 | -0.45 |
| TX Star | NS | NS | 0.26 | 0.13 | 0.39 | 0.13 | 0.08 | 0.25 | 0.27 | 0.74 | - | -0.52 | -0.71 | -0.47 | -0.6 | -0.43 | -0.73 |
| SC Prot | -0.11 | NS | 0.25 | 0.34 | 0.29 | NS | 0.25 | 0.13 | 0.12 | 0.47 | 0.33 | - | 0.57 | 0.23 | 0.16 | 0.41 | 0.29 |
| TX Prot | 0.07 | NS | 0.25 | 0.2 | 0.6 | 0.23 | 0.33 | -0.07 | -0.07 | 0.51 | 0.69 | 0.41 | - | NS | NS | 0.15 | 0.41 |
| SC Fat | NS | 0.07 | NS | -0.09 | 0.12 | 0.27 | -0.09 | 0.57 | 0.58 | 0.64 | 0.53 | NS | 0.09 | - | 0.74 | 0.82 | 0.54 |
| TX Fat | NS | NS | 0.06 | -0.07 | NS | NS | -0.19 | 0.59 | 0.61 | 0.47 | 0.57 | NS | NS | 0.76 | - | 0.58 | 0.7 |
| SC GE | NS | 0.11 | NS | NS | 0.38 | 0.78 | 0.14 | -0.1 | -0.1 | 0.36 | 0.28 | NS | 0.26 | 0.44 | 0.13 | - | 0.62 |
| TX GE | NS | 0.08 | 0.11 | NS | 0.24 | 0.6 | NS | NS | NS | 0.33 | 0.45 | -0.07 | 0.26 | 0.52 | 0.41 | 0.77 | - |
| ^a^ SC, South Carolina environment; TX, Texas environment | | | | | | | | | | | | | | | | | |
| ^b^ DTA, days to anthesis; Hgt, plant height; GNP, grain number per primary panicle; TGW, 1000-grain weight; YPP, grain yield per primary panicle; Amy, amylose; Star, starch; Prot, protein; GE, gross energy | | | | | | | | | | | | | | | | | |
| ^c^ NS, no significance at the *p* = 0.01 probability level | | | | | | | | | | | | | | | | | |

**Figures**

**
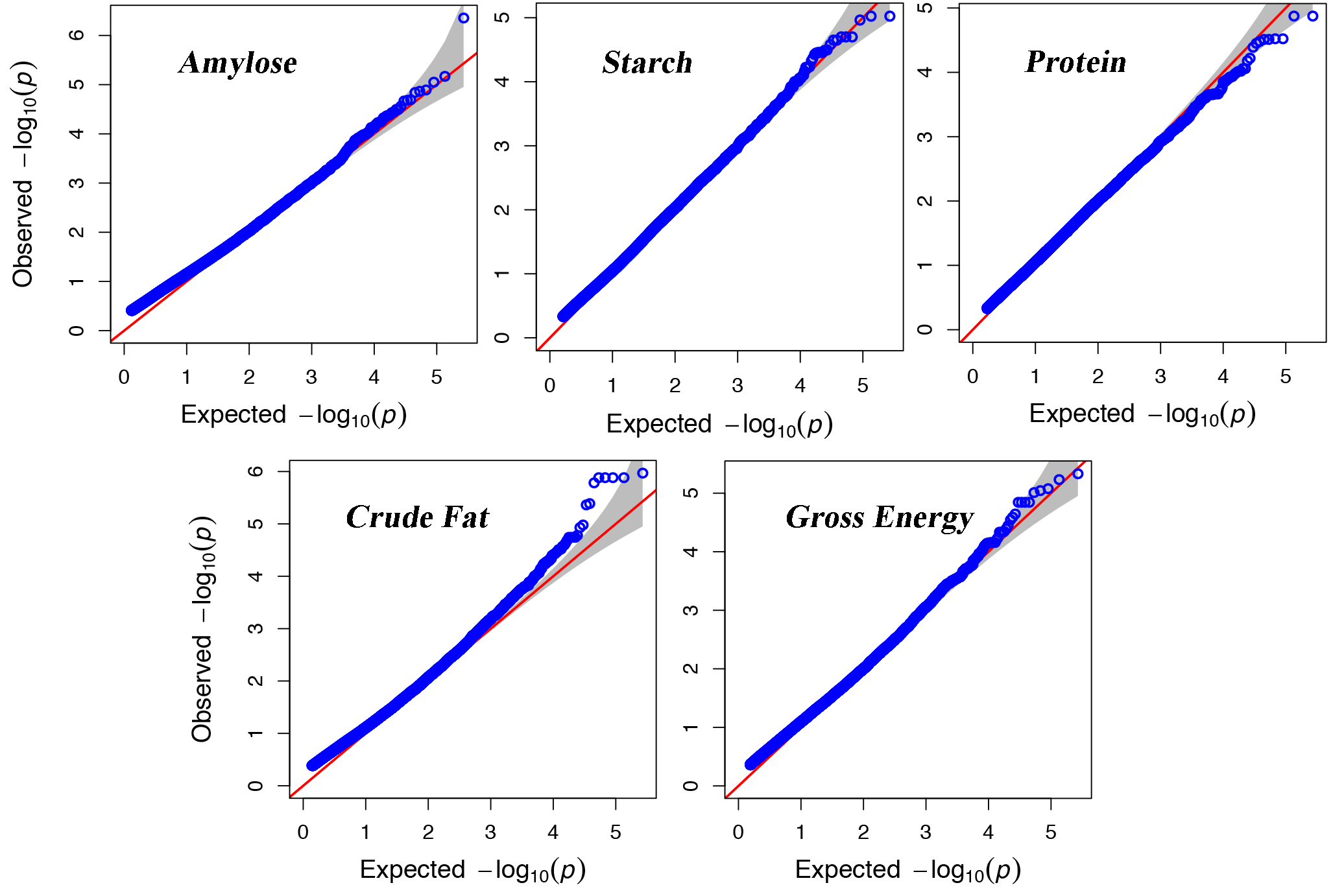
**

**Fig S1.** Quantile-quantile plots displaying expected versus observed significance of each genome-wide SNP (*n* = 268,896) across grain quality traits. Results highlight that the mixed linear model with kinship properly accounted for population stratification. The GWAS was performed using the Genome Association and Prediction Integrated Tool (GAPIT) (Lipka et al., 2012).


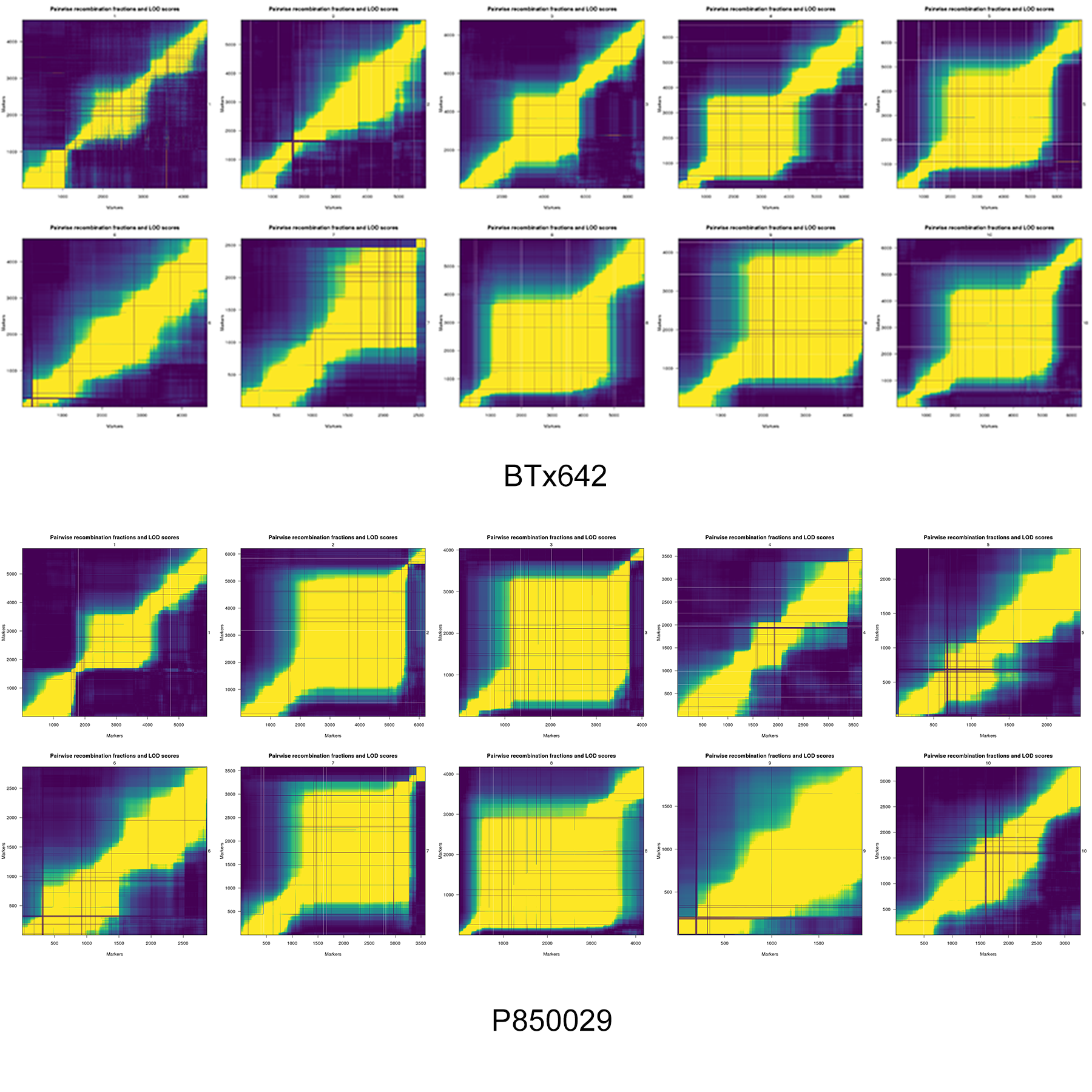


**Fig S2.** Chromosome-wise recombination fraction patterns are shown for the two RIL populations under study.


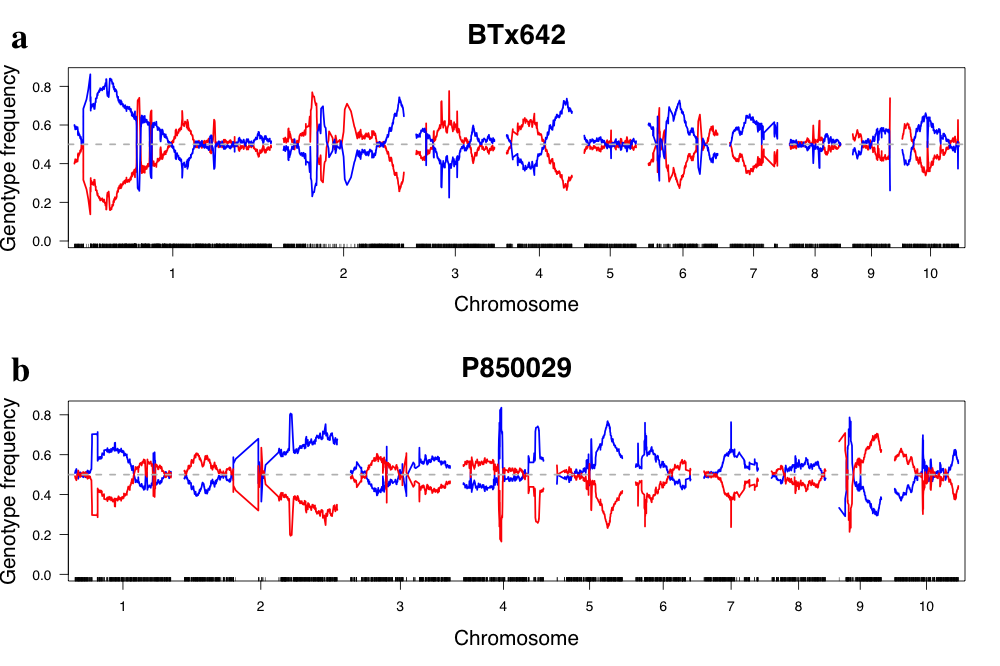


**Fig S3.** Genome-wise allele frequency patterns across recombination bin markers highlight patterns of segregation distortion in both biparental families. **a** Blue, BTxARG-1; Red, BTx642. **b** Blue, BTxARG-1; Red, P850029.


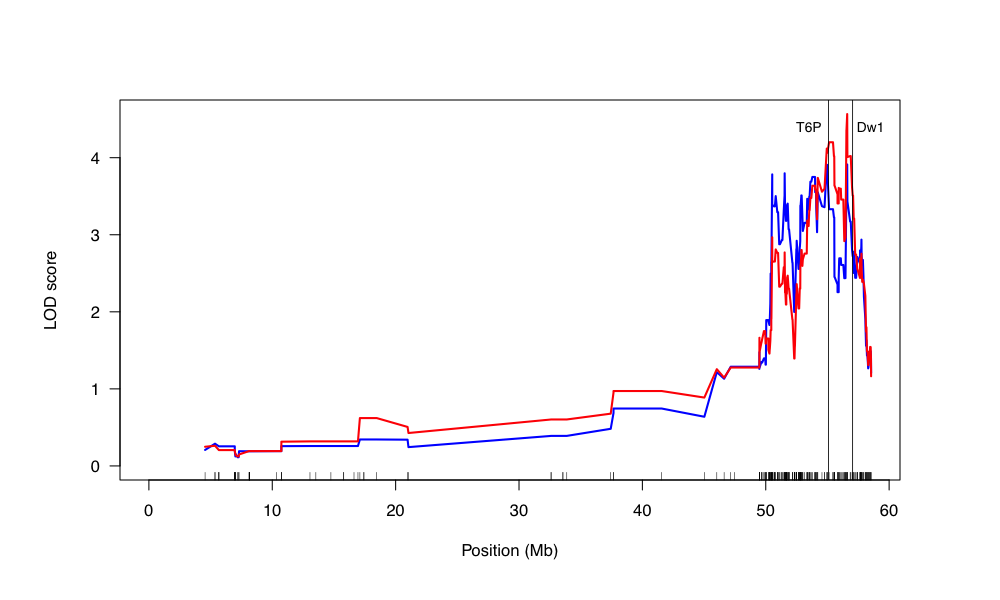


**Fig S4.** Crude protein QTL identified in P850029 on chromosome 9 surrounding the *Dw1* gene. The protein QTL found using the original model (blue) remains significant after including plant height as an additive covariate (blue). Within 20 kb of the bin marker with the maximum LOD score was a putative gene encoding trehalose-6-phosphate (T6P). Black vertical lines mark the middle of each gene.
